# Supplementary material for: Realising the full potential of data-enabled trials in the UK: a call for action
Source: BMJ Open. 2021 Jun 16;11(6):e043906. doi: 10.1136/bmjopen-2020-043906 (PMC8211043; doi:10.1136/bmjopen-2020-043906)
Supplement: Author's manuscript [file bmjopen-2020-043906.draft_revisions.pdf]

BMJ Open

BMJ Open is committed to open peer review. As part of this commitment we make the peer review history of every article we publish publicly available.

When an article is published we post the peer reviewers' comments and the authors' responses online. We also post the versions of the paper that were used during peer review. These are the versions that the peer review comments apply to.

The versions of the paper that follow are the versions that were submitted during the peer review process. They are not the versions of record or the final published versions. They should not be cited or distributed as the published version of this manuscript.

BMJ Open is an open access journal and the full, final, typeset and author-corrected version of record of the manuscript is available on our site with no access controls, subscription charges or pay-per-view fees (http://bmjopen.bmj.com).

If you have any questions on BMJ Open's open peer review process please email info.bmjopen@bmj.com

BMJ Open

Realising the full potential of data-enabled trials in the UK: a call for action

Journal:	*BMJ Open*
Manuscript ID	bmjopen-2020-043906.R1
Article Type:	Communication
Date Submitted by the Author:	05-Dec-2020

Complete List of Authors:	Sydes, Matthew; University College London, MRC Clinical Trials Unit, Institute of Clinical Trials and Methodology<br>Barbachano, Yolanda; MHRA<br>Bowman, Louise; MRC Clinical Trial Service Unit and Epidemiological Studies Unit<br>Denwood, Tom; NHS Digital<br>Farmer, Andrew; University of Oxford, Nuffield Department of Primary Care Health Sciencesd<br>Garfield-Birkbeck, Steph; National Institute for Health Research Evaluation, Trials and Studies Coordinating Centre<br>Gibson, Martin; NWEH<br>Gulliford, Martin; King's College London, UK,<br>Harrison, David; ICNARC,<br>Hewitt, Catherine; The University of York, Health Sciences<br>Logue, Jennifer; Lancaster University<br>Navaie, Will; Health Research Authority<br>Norrie, John; Edinburgh Clinical Trials Unit, University of Edinburgh No. 9, Bioquarter<br>O'Kane, Martin; Medicines and Healthcare Products Regulatory Agency<br>Quint, Jennifer; Imperial College London, Respiratory Epidemiology, Occupational Medicine and Public Health<br>Rycroft-Malone, Jo; Lancaster University; NIHR Health Services & Delivery Programme<br>Sheffield, Jonathan; Formerly CEO NIHR Clinical Research Network; University of Leeds<br>Smeeth, Liam; London School of Hygiene and Tropical Medicine, Epidemiology and Population Health<br>Sullivan, Frank; University of St. Andrews, Division of Population & Behavioural Science; University of Toronto, Department of Family & Community Medicine<br>Tizzard, Juliet; Health Research Authority<br>Walker, Paula; Medicines and Healthcare Products Regulatory Agency<br>Wilding, John; University of Liverpool, School of Clinical Sciences<br>Williamson, Paula; University of Liverpool,<br>Landray, Martin; University of Oxford Nuffield Department of Population Health; University of Oxford Big Data Institute<br>Morris, Andrew; Health Data Research UK<br>Walker, Rhoswyn; Health Data Research UK<br>Williams, HC; University of Nottingham,<br>Valentine, Janet; Medicines and Healthcare Products Regulatory Agency, Clinical Practice Research Datalink

	Data Enabled Trials Workshop Group, And the ; (see member list)
**Primary Subject Heading**:	Health informatics
Secondary Subject Heading:	Evidence based practice, Research methods
Keywords:	Health informatics < BIOTECHNOLOGY & BIOINFORMATICS, STATISTICS & RESEARCH METHODS, Clinical trials < THERAPEUTICS

SCHOLARONE™ Manuscripts

I, the Submitting Author has the right to grant and does grant on behalf of all authors of the Work (as defined in the below author licence), an exclusive licence and/or a non-exclusive licence for contributions from authors who are: i) UK Crown employees; ii) where BMJ has agreed a CC-BY licence shall apply, and/or iii) in accordance with the terms applicable for US Federal Government officers or employees acting as part of their official duties; on a worldwide, perpetual, irrevocable, royalty-free basis to BMJ Publishing Group Ltd ("BMJ") its licensees and where the relevant Journal is co-owned by BMJ to the co-owners of the Journal, to publish the Work in BMJ Open and any other BMJ products and to exploit all rights, as set out in our licence.

The Submitting Author accepts and understands that any supply made under these terms is made by BMJ to the Submitting Author unless you are acting as an employee on behalf of your employer or a postgraduate student of an affiliated institution which is paying any applicable article publishing charge ("APC") for Open Access articles. Where the Submitting Author wishes to make the Work available on an Open Access basis (and intends to pay the relevant APC), the terms of reuse of such Open Access shall be governed by a Creative Commons licence – details of these licences and which Creative Commons licence will apply to this Work are set out in our licence referred to above.

Other than as permitted in any relevant BMJ Author's Self Archiving Policies, I confirm this Work has not been accepted for publication elsewhere, is not being considered for publication elsewhere and does not duplicate material already published. I confirm all authors consent to publication of this Work and authorise the granting of this licence.

For peer review only

**Title:**	**Realising the full potential of data-enabled trials in the UK: a call for action**
**Running title:**	Data enabled trials in the UK
**Version and date:**	Version 1.01; (27-Nov-2020) -- Submitted version (ScholarOne edits)
**Tables and Figures:**	2 Tables<br>7 Boxes<br>0 Figures<br>0 Supplementary tables<br>0 Supplementary figures<br>0 Appendices

Authors:

Sydes, Matthew (0000-0002-9323-1371)
MRC Clinical Trials Unit at UCL, Institute of Clinical Trials and Methodology, UCL, London, UK
& HDR UK

Barbachano, Yolanda
Medicines and Healthcare products Regulatory Agency (MHRA)

Bowman, Louise (0000-0003-1125-8616)
Clinical Trial Service Unit and Epidemiological Studies Unit, Nuffield Department of Population Health, University of Oxford, Oxford

Denwood, Tom (0000-0002-7337-2425)
NHS Digital

Farmer, Andrew (0000-0002-6170-4402)
Nuffield Department of Primary Health Care Sciences, University of Oxford.

Garfield-Birkbeck, Steph (0000-0002-3181-2840)
National Institute for Health Research Evaluation, Trials and Studies Coordinating Centre

Gibson, Martin (0000-0002-1331-1524)
NWEH Ltd

Gulliford, Martin (0000-0003-1898-9075)
Kings College London, London

David Harrison (0000-0002-9002-9098)
Intensive Care National Audit & Research Centre (ICNARC), London, UK

Hewitt, Catherine (0000-0002-0415-3536)
York Trials Unit, Department of Health Sciences, University of York

For peer review only - http://bmjopen.bmj.com/site/about/guidelines.xhtml

Logue, Jennifer (0000-0001-9549-2738)
Lancaster University

Navaie, Will
Health Research Authority

Norrie, John (0000-0001-9823-9252)
Edinburgh Clinical Trials Unit, University of Edinburgh

O'Kane, Martin
Medicines and Healthcare products Regulatory Agency (MHRA)

Quint, Jennifer (0000-0003-0149-4869)
NHLI, Imperial College London, UK

Rycroft-Malone, Jo (0000 0003 3858 5625)
Lancaster University & NIHR Health Services & Delivery Programme

Sheffield, Jonathan
Formerly CEO NIHR Clinical Research Network; University of Leeds

Smeeth, Liam (0000-0002-9168-6022)
London School of Hygiene and Tropical Medicine

Sullivan, Frank (0000-0002-6623-4964)
Division of Population & Behavioural Science, University of St. Andrews
Department of Family & Community Medicine, University of Toronto

Tizzard, Juliet
Health Research Authority

Walker, Paula
Medicines and Healthcare products Regulatory Agency (MHRA)

Wilding, John (0000-0003-2839-8404)
Department of Cardiovascular and Metabolic Medicine
Institute of Life Course and Medical Sciences
University of Liverpool

Williamson, Paula (0000-0001-9802-6636)
University of Liverpool

ϕ Landray, Martin (0000-0001-6646-827X)
Nuffield Department of Population Health, University of Oxford, Oxford, UK
Health Data Research UK, University of Oxford, Oxford, UK
NIHR Oxford Biomedical Research Centre, Oxford University Hospitals NHS Foundation Trust, Oxford, UK

ϕ Morris, Andrew (0000-0002-1766-0473)
Health Data Research UK

For peer review only

ᶲ Walker, Rhoswyn
Health Data Research UK

ᶲ Williams, Hwyel (0000-0002-5646-3093)
Professor of Dermato-Epidemiology, University of Nottingham

ᶲ Valentine, Janet
Clinical Practice Research Datalink

And the Data Enabled Trials Workshop Group

**Contributors:**	See list of author contributions at end of document
**Trial identification:**	N/A
**Correspondence:**	Matthew Sydes m.sydes@ucl.ac.uk
**Sponsor:**	N/A
**Funding &support:**	The workshop was co-funded by NIHR Health Technology Assessment Programme, CPRD and HDR UK
**Competing Interests**	All authors have completed the ICMJE uniform disclosure form at www.icmje.org/coi_disclosure.pdf. Please see the end of the manuscript for full list of author disclosures.
**Transparency declaration:**	All authors affirm that the manuscript is an honest, accurate, and transparent account of the study being reported; that no important aspects of the study have been omitted; and that any discrepancies from the study as planned (and, if relevant, registered) have been explained
**Ethical approval:**	(Not required)
**Data sharing:**	(No new data)

For peer review only

	Section	Words	Cumulative
**Word Count:**	Abstract	333	n/a
	Introduction	491	491
	Findings > Design	707	1198
	Findings > Recruitment	878	2076
	Findings > Conduct	587	2663
	Findings > Outcomes	831	3494
	Findings > Analysis	627	4121
	Discussion	1876	5997
	**Total**	**5997**	

(Word counts cover the body text of each section and exclude the headings and the ending notes)

For peer review only

ABSTRACT

Rationale: Clinical trials are the gold standard for testing interventions. COVID-19 has further raised their public profile, and emphasised the need to deliver better, faster, more efficient trials for patient benefit. Considerable overlap exists between data required for trials and data already collected routinely in electronic healthcare records (EHR). Opportunities exist to utilise these in innovative ways to decrease duplication of effort, and speed trial recruitment, conduct and follow-up.

Approach: NIHR), HDR UK and CPRD co-organised a national workshop to accelerate the agenda for "data-enabled clinical trials". Showcasing successful examples and imagining future possibilities, the plenary talks, panel discussions, group discussions and case studies covered: design/feasibility; recruitment; conduct/follow-up; collecting benefits/harms; and analysis/interpretation.

Reflection: Some notable studies have successfully accessed and used EHR to identify potential recruits, support randomised trials, deliver interventions and supplement/replace trial-specific follow-up. Some outcome measures are already reliably collected; others, like safety, need detailed work to meet regulatory reporting requirements. There is a clear need for system interoperability and a "route map" to identify and access the necessary datasets. Researchers running regulatory-facing trials must carefully consider how data quality and integrity would be assessed. An experience-sharing forum could stimulate wider adoption of EHR-based methods in trial design and execution.

Discussion: EHR offer opportunities to better plan clinical trials, assess patients and capture data more efficiently, reducing research waste and increasing focus on each trial's specific challenges. The short-term emphasis should be on facilitating patient recruitment and for post-marketing authorisation trials where research-relevant outcome measures are readily collectable. Sharing of case studies is encouraged. The workshop directly informed NIHR's funding call. ambitious data-enabled trials at scale. There is the

For peer review only - http://bmjopen.bmj.com/site/about/guidelines.xhtml

opportunity for the UK to build upon existing data science capabilities to identify, recruit and monitor patients in trials at scale.

For peer review only

For peer review only - http://bmjopen.bmj.com/site/about/guidelines.xhtml

INTRODUCTION

Clinical trials will continue to be the gold-standard for testing pharmacological, behavioural and policy interventions for the foreseeable future. The advent of the COVID-19 pandemic has raised the profile and importance of trials with the UK public.[1] For a large majority of conditions, late-phase randomised controlled trials are commonly long-term endeavours which are increasingly expensive, particularly Industry-led trials. There is considerable overlap between the data that trialists need to assess impact of interventions on patients and what is already routinely collected in patient's health records. These routinely-routinely collected data are reflected in local, regional or national datasets, collectively labelled here as "electronic health records" (EHR) (**Box-1**). It is equally self-evident that safely making these routinely-routinely collected data available to researchers for purposeful access could decrease duplication of effort, reduce costs and may speed answers from some trials. The value of EHR in clinical trials is increasingly recognised by many organisations, including the UK Government as highlighted in the Life Sciences Industrial Strategy.[2,3]

The National Institute for Health Research (NIHR), Health Data Research UK (HDR UK) and the Clinical Practice Research Datalink (CPRD) (**Box-2**) brought together UK researchers for a one-day workshop in Oct-2018 to discuss how clinical trials might undergo a step-change in the UK through being "data-enabled". "Data-enabled trials" were defined for the workshop as introducing efficiencies in the following ways: improved study planning and feasibility assessments; more efficient recruitment, facilitated by accessing EHR to identify potential participants; efficient assessment of treatment benefits and potential harms by directly capturing routine EHR into the trial dataset; and more efficient study analyses and interpretation, using standard EHR variables. Many lessons from the workshop are also relevant to clinical trials more broadly.

For peer review only - http://bmjopen.bmj.com/site/about/guidelines.xhtml

Informed by this workshop, NIHR commissioned a cross-programme call for "Data-enabled trials"[4]. **Table-1** contains a list of data-enabled trials that were funded as a result of this call.

While there is recognition that challenges exist to accessing and using all sources of EHR, great strides have already been made realising the potential for using available EHR in innovative ways to support the delivery of patient consented studies. The workshop's spirit was therefore to focus on what had already been achievable, and to consider imaginable solutions to current challenges and further possibilities that could be developed in the future at scale. Speakers and facilitators were asked "to demonstrate existing capability and opportunities in the UK for undertaking data-enabled clinical trials, in particular showcasing the use of routine data to support different components of trial design and delivery, and regulatory considerations." The workshop, which was attended by researchers from around the UK, included plenary talks, panel discussions, group discussions and the presentation of selected case studies. The elements were broadly grouped as: Design and feasibility; Recruitment; Conduct and follow-up; Collecting key data on benefits and harms; and Analysis and interpretation. Here, we summarise and update some key issues that emerged in these areas in order to inform future discussions and research activities.

For peer review only

FINDINGS

Design and feasibility

Assessing the potential sufficiency of accrual is key to the planning and funding of all trials. Recruitment can be challenging both in prevalent and incident clinical conditions, particularly where potential participants are managing their chronic conditions in an environment away from active researchers. Robust and scalable processes to estimate the number of potential participants that might be suitable for a particular study need to be established.

Historically, complex care pathways made it difficult to estimate, and then locate, potential trial participants, because they might enter the systems in a number of ways. Use of evidence from the EHR, where these are available, enables a more realistic estimate of the achievable sample size within specific geographical regions, based on demographic and clinical information within EHRs. Key eligibility criteria such as demographic information, medication dose, laboratory results, timing of clinical events and comorbidities within the EHR allow rapid estimates of the number of potentially eligible people that can be flagged as suitable for a trial.

In the UK, general practitioners (GPs) are the gatekeepers to the NHS, and, as such, primary care records hold information that includes demographic and clinical data derived from consultations within primary care as well as secondary care visits and from other sources such as laboratory tests. Primary care records, covering a patient's medical history, are therefore a rich resource to carry out feasibility assessments and to locate patients for studies that might take place in a range of settings. Timeliness of data is important. The primary care data that CPRD receives, encompassing 25% of UK population, is updated daily, and it forms the basis for feasibility assessments CPRD routinely carries out for academic and industry led trials.[5] To further advance the use of EHR in supporting clinical trials, the NHS DigiTrials Health Data Research

For peer review only - http://bmjopen.bmj.com/site/about/guidelines.xhtml

Hub for Clinical Trials was established by HDR UK in 2019 to facilitate feasibility assessments in England, initially using secondary care Hospital Episode Statistics (HES).[6,7]

The usual method of locating patients for many studies involves manual searches of patient records at each GP practice. The COPE study[8], a patient-consented, longitudinal cohort study, was one of the first studies in the UK exploring the use of EHR as a means of locating specific patients for enrolment into a study. The study, which sought to identify patients with chronic obstructive pulmonary disease (COPD) with prior exacerbation events (**Box-3**), demonstrated that it is possible to use a primary care EHR database to screen, locate and recruit participants for research. This method "provides access to a cohort of patients while minimizing input needed by GPs and allows researchers to examine healthcare usage and disease burden in more detail and in real-life settings".[8] CPRD carried out a centralised search for eligible patients in its database of pseudonymised patients, across a defined geographical region and GP's in CPRD's network were supplied with a list of potentially eligible patients based on the study inclusion and exclusion criteria. The GP then carried out a clinical review and invited potentially suitable patients to attend specific centres in which recruitment and collection of bespoke data could be completed. This intermediate clinical validation step involving the GP confirming a patient's eligibility, both facilitated locating high quality patients and maintained patient confidentiality.

A large proportion of trials fail to recruit to time and target,[9] and the funding wasted on these efforts may be better invested in modest pre-trial efforts to determine likely feasibility. Assessing potential numbers of patients can reasonably be done with near-real-time, longitudinal EHR (likely primary care datasets) or an administrative EHR record, like HES, which is cross-sectional and broad, but has a time-delay. Given the demonstrated value of using a EHR evidence-based approach to assessing feasibility, research funding bodies will need to give careful consideration to how such work-up activities can be supported as dedicated funding for research usually starts only after the work to assess feasibility has been attempted. Key points to emerge for study planning using EHR were the importance of using a validated algorithm to

For peer review only - http://bmjopen.bmj.com/site/about/guidelines.xhtml

search the data and the benefits of incorporating a clinical review to further refine the specificity of the search. These approaches can result in a much higher proportion of suitable and engaged patients presenting for detailed eligibility assessment at study sites.

Recruitment

Recruiting patients to research studies should be a standard part of routine practice. The majority of UK hospital trusts engage in research activity and this is increasingly true in primary care with 40% of GP practices in England participating in research in 2018/19.[10] However, for most practitioners, there is limited time to spend with patients, and often not enough time to explain research so lower burden approaches must be found. [11,12] Searches of GP patient records for patients potentially suitable for clinical studies isn't new. However, this is usually done by searching right through GP patient records on a local practice basis, which can be a burden for practice staff and introduce variability in interpretation of selection criteria. Centralised patient eligibility searches, such as those carried out by CPRD and North West eHealth, reduce the burden on practice staff by supplying the practice a list of potentially eligible patients located through standardised database searches against protocol inclusion and exclusion criteria.

Large-scale use of EHR for patient searches must take into account information governance regulations and the need to maintain patient confidentiality. One approach is to search pseudonymised patient data, where a patient's identity is unknown to the researchers, as is the case with CPRD recruitment services. Potentially suitable patients are re-identified by their GP, thereby maintaining confidentiality between the patient and the clinician. In some cases, specific regulatory approvals may be granted by to individual researchers to obtain access to identifiable EHR patient data in order directly invite them into a clinical trial. Alternative methods for recruiting patients into prospective trials include establishing a cohort of patients or volunteers who are willing to take part in trials, such as the NHS Scotland SHARE scheme[13] and COVID-19 vaccine trials portal,[14] and pre-consenting trial participants to be re-approached about other studies, such as those following on from the INTERVAL trial of blood donation timing.[15]

For peer review only - http://bmjopen.bmj.com/site/about/guidelines.xhtml

EHR are a tool that can be used to speed the pairing of willing patients with suitable trials.[16] DECIDE is a trail-blazing randomised pragmatic clinical effectiveness trial, involving a three-way partnership between academia, industry and CPRD, which recruited patients with Type-2 diabetes in a routine primary care setting, randomised to the licenced intervention or standard of care.[17] The study involved centralised searches of CPRD's primary care data to enable GPs in the CPRD network across all four UK nations to readily enrol suitable patients into the study when they presented for their routine diabetes checks. The novelty of this pragmatic clinical effectiveness study was that the study clinical data was captured directly from the patient's primary care EHR and flowed into CPRD's interventional research services platform (IRSP). The trial including randomisation was remotely managed via this central digital platform. The EHR derived study data was supplemented by electronic patient-reported outcomes collected by patients remotely logging into the IRSP. **Box-4** summarises the key lessons from DECIDE, using real-time EHR to facilitate recruitment and trial management. The DECIDE study demonstrates how it is possible to recruit patients across the UK into a randomised trial involving real-world Type-2 diabetic patients, based on changing laboratory HbA1c readings using dynamic data searches on primary care EHR data. This approach of using primary care data for time-bound patient recruitment, offers great potential and generalisability for Phase-II to Phase-IV patient recruitment, independent of relying on busy clinicians identifying patients presenting at clinic visits.

The majority of clinical trial protocols involve inclusion or exclusion variables that are time-dependent, for example timeframe from diagnoses, procedures or prescriptions. Furthermore, many trials will have a limited window of opportunity during which recruitment can take place for any individual potential participant. If patients are identified too late, the research invitation could arrive after it stopped being relevant; anecdotally, such irrelevant invitations have been confusing and upsetting to vulnerable populations. In these circumstances, the EHR source used to located suitable patients for trial recruitment must contain patient information that is both current and dynamic.

For peer review only - http://bmjopen.bmj.com/site/about/guidelines.xhtml

Primary care data can provide a longitudinal and timely resource for locating patients suitable for both incident and prevalence trials. For example, CPRD's primary care records are updated daily and include hospital discharge information, so the system lends itself well to locating patients for trials taking place in primary or secondary care. GPs can also be alerted through system 'pop-ups' or dynamic daily refreshed patient lists, as for the DECIDE trial, which can support recruitment into incident trials for patients who present at the GP practice with acute or unmanaged conditions.

HES data can also be used to locate patients who have visited hospital and may be suitable for a trial. The ORION-4 trial is using a combination of HES data searches and patient questionnaires to recruit 15,000 patients from across the UK with atherosclerotic cardiovascular disease.[18] However, HES data has its limitation as it may only be available some months (often 3 months) after a patient hospital visit, it does not include a patient's full medical history and may not include all potential patients, such where the condition was not severe enough to require a hospitalisation eg mild stroke. Some hospitals have developed their own EHR systems that support recruitment of patients presenting with acute conditions. However, there are currently no cross-hospital, inter-operable electronic systems in use in secondary care that support dynamic recruitment.

Conduct and follow-up

Access to routine EHR for individuals participating in a trial to monitor their important and relevant health outcomes has been a fundamental challenge for many trials that have attempted access late in the trial. This has often involved a lengthy application process via a changing approval system, frequently resulting in a considerable lag between requesting and accessing data. Many researchers have persisted, recognising the huge potential for efficient use of EHR in the conduct and follow-up of trials, particularly large-scale national trials.

For peer review only - http://bmjopen.bmj.com/site/about/guidelines.xhtml

ASCEND[19,20] was a UK-wide trial in 15,500 people with diabetes, evaluating the effect of aspirin and omega-3 fatty acids on the risk of cardiovascular events. The trial, whose key design features are summarised in **Box-5**, used mail-based methods for recruitment, intervention delivery and follow-up, thereby avoiding the need for dedicated study sites. Initially, follow-up was by a paper-based questionnaire, but, in the latter years of the trial, an online form was available for follow-up for those who preferred. Towards the end of the 7.5 year follow-up period, access to HES data was granted, which allowed missing outcome data to be recorded for participants who had not returned questionnaires. Preliminary unpublished comparisons in ASCEND between the questionnaire data and the EHR data showed good concordance, with analyses of the primary trial outcomes producing similar results irrespective of the data source used. [Personal communication]

Although the trial was beneficial for patients avoiding the need to travel to trial sites, it was labour-intensive for the study team, involving mailing and phoning thousands of participants and their GPs. Considerable efficiencies could have been possible if the HES data had been used as the main source of outcome information from the outset. Further benefits could be derived by accessing primary care data, as some cardiovascular events do not require hospitalisation and are consequently not captured in the HES data. The HES dataset is usually at least 3 months old. However, the 'rawer' form of HES, called Secondary Uses Service (SUS+) is being used in the RECOVERY trial to enable faster, albeit *in extremis,* assessment of health outcomes for patients with severe COVID-19 infection.

EHR can be used to assess the short-and long-term impact of an intervention on health outcomes of individual patients at participating sites. This can be through a patient-level intervention or via a randomised cluster trial where the intervention is applied at a site level. One such example is the REDUCE cluster randomised trial embedded with the CPRD GP practice research network which used a digital intervention versus standard of care to attempt to reduce unnecessary use of antibiotics for respiratory

For peer review only - http://bmjopen.bmj.com/site/about/guidelines.xhtml

infections in primary care. In addition to using the EHR to monitor prescribing rates, EHR data was used to monitor patient health outcomes in the intervention and standard of care GP practice arms of the trial.[11] Findings from ASCEND[19,20], COPE[8] and DECIDE[11] support the advantages gained for data collection and follow-up by using EHR from the outset for patients recruitment.

The Office for National Statistics system has flagged people as participating in each of around 200 approved trials so that mortality data could be provided to the trial in due course. All EHR systems need to include this ability to flag for participation in a specific trial. Without flagging, trialists and EHR providers need to go through the linkage process. This increases costs against limited budgets, is disproportionately effortful and exposes a repeated risk of linkage errors. Both Public Health England National Cancer Registration and Analysis Service (NCRAS) and NHS Digital are piloting approaches to trial flagging.

Collecting outcome data on benefits and harms

Access to timely data is key for any trial, and trialists must be able to report on safety and key outcome measures in a suitable timescale, including for the final analyses and for Data Monitoring Committee review of interim analyses. Trials must meet the stringent regulatory timelines set out in UK law, which includes reporting to the competent authority, appropriate suspected unexpected serious adverse reactions (SUSAR), within short timelines that starts when the sponsor of the trial receives the information from the clinical site.

The timely capture and reporting of certain safety outcome measures are critically important for the Clinical Trials of Investigational Medicinal Products (CTIMP) trials run under the clinical trials regulations, where a subset of Serious Adverse Events (SAE) must be reported to the regulator within a short, prescribed timescale of 7 or 15 days. Researchers must quickly determine whether the SAE is a “reaction” (SAR) and, if so, whether this reaction is an “unexpected” (SUSAR) according to clearly-defined Reference Safety Information. SAR and SUSAR are more likely in some treatment settings e.g. cancer chemotherapy

For peer review only - http://bmjopen.bmj.com/site/about/guidelines.xhtml

trials. “Seriousness” definitions include, among others, “hospitalisation” or “prolongation of hospitalisation” which may or may not be captured across different EHR systems.

Frequency of updates to routine EHR data depends on where in the system the data are collected. Primary care records, such as those received by CPRD, are updated daily, but administrative-focused EHR datasets, such as HES, may provide only a snapshot of data from some months ago. Trials using only periodically-updated, routinely-collected EHR generally cannot rely on these data for reporting purposes in regulatory timescales, in particular for safety monitoring. It is a key responsibility of researchers to understand the strengths and limitations of any EHR data source, including its suitability to meet reporting requirements. A future involving interconnected real-time systems would allow investigators to reliably detect and report appropriate events.

The Salford Lung Studies were two industry-sponsored, late-phase RCTs which were the first studies in the world to have evaluated the effectiveness of a pre-licenced medicine in a real-world setting. The study capitalised on access to primary care EHR, as well as community pharmacy data and electronic secondary care patient records embedded the regional hospitals. Altogether 7200 patients with COPD or Asthma were monitoring in near real-time for safety and outcomes using city wide linked EHR.[21 22] The study team met with the regulators to agree the outcome measure data and safety alerting and reporting systems, which were predicated entirely on capture of routine data from near real-time primary and secondary care systems. As outlined in **Box-6**, the studies successfully demonstrated that it is possible to run complex large-scale trials with configurable near real-time safety monitoring systems that meet regulatory standards

It is important to check up-front in efficacy and effectiveness trials that may be used for regulatory submission whether routinely-collected EHR would be accepted as source data by the relevant regulatory authorities. The Medicines and Healthcare products Regulatory Agency (MHRA) now considers using

For peer review only - http://bmjopen.bmj.com/site/about/guidelines.xhtml

source data directly from each hospital's EHR standard following digitalisation of health records. GCP inspections performed by the MHRA in the UK are performed against the requirements of the UK Clinical Trials Regulations, irrespective of whether the source data is electronic or paper in format. The MHRA recognises that there is no standardised method of recording clinical source data, especially in international multi-centre trials.[Personal communication] Variations across sites include how many electronic systems make up the EHR, and how clinical data are reported through local laboratory electronic systems. It is therefore recommended each sponsor reviews the type of data required for their trial and how it would be recorded at site, including the various electronic systems that capture the source data and whether they are fit for purpose.

The source data and its underpinning EHR system needs to follow the ALCOA+ principles to be attributable, legible, contemporaneous, original and accurate, as well as complete, consistent, enduring and available.[23] This ensures the data are reliable and that the electronic system is fit for its intended purpose. This should include appropriate access levels for investigator site trial teams, sponsor representatives and Government Regulators, and is supported by the ICH E6 R2 Addendum. Careful work is required to ensure that systems which collate data from multiple healthcare systems (eg HES or registry data) follow these principles. Review of the data on regulatory inspection by the MHRA includes assessing whether the trial data can be reconstructed, and that the above requirements are met. Reconstruction includes assessing whether the system has an associated audit trail verifying who completed assessments and when, and the ability to reconstruct any changes to the source data. Source data verification performed during MHRA inspections is always done on a risk-based approach, with a focus on the critical data for the trial and compliance with the clinical trials regulations, recognising that EHR systems are often designed with standard care not clinical trials in mind. The same risk-based approach should be taken by sponsors auditing their own trial data.

For peer review only - http://bmjopen.bmj.com/site/about/guidelines.xhtml

Analysis and interpretation

Many researchers desire collecting as much data as possible for their trial through routine EHR sources, thereby minimising the collection of data through trial-specific means. Reviewing metadata or de-identified samples of data, broadly representative of patients that could join a future trial, is recommended to determine which datasets include suitable variables to monitor health outcomes from EHR sources. In the case of cancer, Public Health England has developed Simulacrum to imitate some of the data held by their National Cancer Registration and Analysis Service.[24] The Health Data Research Innovation Gateway has been initiated by HDR UK as an open repository of data with associated metadata, to accelerate discovery and access to existing health data research resources.[25] Some argue that a common repository of data quality checks may be useful for researchers running data-enabled trials. Carrying out data utility assessments are a practical method of determining where EHR could substitute for trial-specific collection in future trials.[26,27]

Any one EHR dataset may not collect all the necessary information in a timely and consistent manner that can be used to evaluate health outcomes across all trial arms. **Box-7** sets out some of the lessons learned during the SANAD-II trial.[28,29] This trial for patients with epilepsy found that seizures, key to any epilepsy core outcome set, had not been well recorded in the accessible EHR sources. Initiatives such as the COMET initiative are seeking to develop a set of core outcome measures that are suitable for randomised trials, audit, research and routine care. Researchers should strive to make core outcome sets as similar as possible to both the information required in routine practice and the key outcomes needed by regulatory reviewers.

Patients' records held as EHR datasets at hospital are rich in specific areas but lack the wider medical history. Patients' records at GP surgeries capture the broader, longitudinal medical history. Registry datasets may also have a great depth of healthcare data but in a narrow patient sub-group. Central administrative datasets collating information across healthcare systems may be wide but not deep.

For peer review only - http://bmjopen.bmj.com/site/about/guidelines.xhtml

Therefore, it may be necessary to use multiple datasets to analyse trial outcomes and these datasets may need to be linked. Well established processes exist to link data to trials but these can cause delays due to governance and data access issues.

Data linkage can be used to validate data collection on an individual trial participant and can support assessment of generalisability and external validity. Use of alternative data sources could address any concerns about the representativeness of patients who were not invited to participate in a trial (e.g. trial not open in the area) and checking that the participants are broadly representative of the target population. An example of this is where primary data were used to validate the outcomes of the real-world Salford Lung Studies, in the broader UK population thereby ruling out any bias that may be introduced due to a regional Hawthorn effect.[30] For treatments that are widely available outside of the research setting, researchers may assess external validity or representativeness of the patient populations if data can be requested for an equivalent non-randomised anonymised cohort of patients.[11]

For trials that combine multiple sources of data, trialists must pre-specify what will be done in the instance of discrepancy between sources: which source would take primacy, who makes the decision, how checking will be done, and whether there is any opportunity to correct one of the data sources, if required, in a manner consistent with data protection regulations about holding accurate data.

It is critically important to ensure that the correct EHR variables are both well understood and used in the right way to calculate the relevant outcome measures. Using multiple sources with their current differences in terminology and structure could increase the likelihood of error.

For peer review only

DISCUSSION

NHS datasets provide a record of population-level health data and afford rich opportunities for improving health through evidence-based medicine. There is a recognition that the UK can grasp these opportunities and address the challenges required to usher in a new generation of more efficient and cost-effective clinical trials. Many of these trials will be "data-enabled trials", with routinely-collected EHR driving their design and feasibility, their screening and recruitment and their management and analysis. Others will use EHR for more established practices such as long-term participant follow-up. The workshop demonstrated that EHR can readily be used to assess trial feasibility and to facilitate patient recruitment from Phase-II to Phase-IV. However, attendees sensed that the short-term emphasis for conducting "fully data-enabled trials" should be on licenced treatments in different populations or for new indications, where outcome measures are readily collectable from the EHR. Trials like the Salford Lung Studies and DECIDE serve as vanguards demonstrating the application of real-world evidence in fully data-enabled clinical trials.[17,21,22]

EHR data are collected in different formats and frequency depending on their purpose and provenance. There are multiple EHR–collating-and-holding bodies within the UK, but no one body can provide access to all sources of EHR data. This situation results in a complicated web of access routes to datasets, differing in scope, method of collection, currency of data, quality controls, availability of metadata and connection to additional linked information. It is currently estimated that fewer than 5% of trials in the UK successfully access routinely-collected datasets to support trial execution,[31] much lower than the proportion of trials planning to access such data at the grant application stage.[32] Therefore, more is clearly needed to increase confidence and capabilities in the use of routine health data across the UK clinical trials community. In the short-term, route maps depicting these dataset details and access methods would be welcomed. In the long-term, the trials community would be keen to see intra-operable EHR systems providing simple access to up-to-date datasets to facilitate trials: intra-operability and reusability emerged repeatedly as themes throughout the workshop. There are complex and varying coding systems across the

For peer review only - http://bmjopen.bmj.com/site/about/guidelines.xhtml

various healthcare practice settings for which clarity is required. The UK Secretary of State has been aware of the need for better data standards in healthcare and has started to put NHS quality data into dashboards.[33] Many data items are held in free-text so sophisticated natural language processing (NLP) approaches will be required to extract coherent, targeted information, whilst upholding confidentiality standards. The Health Data Research UK's Health Data Research Innovation Gateway have has progressed towards making more accessible the datasets routinely available datasets more accessible for a range of research uses.[34] although initially this resource is more likely to facilitate re-use of data from trials rather than supporting recruitment and conduct of new trials.

Gaining the necessary permissions to access EHR data causes challenges for many researchers, particularly when the interpretation of patient consent has changed over time with the strengthening information governance requirements. Consent from trial participants to access EHR data is taken in many trials only with the intention of accessing long-term outcome measures, so researchers may not submit the relevant applications until the linked data are needed, late in the trial. Unfortunately, expectations around consent might have changed by this time point, complicating the process. Researchers are therefore strongly recommended to access EHR data from early in the trial and for trial consent forms to prospectively cover broad and enduring consent for access to routine EHR as a matter of course in order to facilitate research.[35]By embedding the EHR from the design stage, with appropriate consent, data-enabled trials avoid this issue. A more streamlined approach to governance with greater consistency and clarity in terminology and requirements for enduring patient consent for research may facilitate future data access for all trials. Where additional data are required and collected for a clinical trial, there may be potential to augment the patient's EHR with this information, such as results of genetic or genomic tests or patient-reported outcome measures.

Clinical trials are expensive and slow to plan and complete, and it is necessary to reduce research waste through all possible methods.[36-39] Trial design takes at least 6 months, yet is often insufficiently resourced,

For peer review only - http://bmjopen.bmj.com/site/about/guidelines.xhtml

relying on "own account" work taken at the risk of the sponsor (usually a higher education institute) prior to grant award. A substantial proportion of trials are delayed or do not reach their recruitment target;[9,40] and a considerable number require at least one protocol amendment.[41] EHR offer opportunities to better plan clinical trials by characterising the populations and their treatment or diagnostic pathways as well as assessing the trial feasibility based around specific geographies.[8] The proportion of patients approached and recruited to trials also varies according to different demographics such as ethnicity or age.[42,43] Using EHR data to identify specific patient pools will enable trials in under-served populations or where disease burden is the greatest, thereby democratising research and increasing the potential recruitment efficiencies.[44] Increasing the representativeness of trial participants also enhances external validity of the findings, especially as guidance such as the INCLUDE framework is now available on the issues to consider to be more inclusive on attributes such as ethnicity.[45] UK-wide representative population coverage, through CPRD, has demonstrated the proof of principle of successfully recruiting a demographically representative patient population into the pragmatic DECIDE in Type-2 diabetes trial, through centralised primary care patient data eligibility searches [Personal communication]. Virtually all of the UK population is registered with a GP practice and GPs are gatekeepers to the NHS, often managing chronic conditions that have been diagnosed in secondary care. With its rich, longitudinal medical history, primary care data therefore represent an excellent source of data to locate suitable patients for trials based in both primary and secondary care settings. A strong desire is a single resource which brings together all primary care data within and across all four UK nations.

Validation of the choice of EHR codes used in patient cohort searches enables greater specificity of patient searches, leading to higher patient conversion rates into and less time lost by patients and trial teams at the trial screening stage.[Personal communication] Clinical validation of potentially suitable patients initially flagged by EHR searches, further refines the patient search, meaning that the patients approached to take part are more likely to pass eligibility screening, which improves the efficiency of patient recruitment. Assessment of the variables contained within the EHR at the trial design stage enables a

For peer review only - http://bmjopen.bmj.com/site/about/guidelines.xhtml

better understanding of which variables can be obtained from routinely-collected data, removing duplication of collection. Moving from only trial-specific data collection to more widespread use of EHR within trials, should improve the cost-effectiveness of trial funding. Double-blinding and placebo-controls are more difficult aspects of conduct to operationalise in routine EHR settings, but may be offset by robust data, careful protocols and objective outcome measures. Each trial's risk assessment should be clear on any risks associated with the use of routine EHR data. Formal regulatory advice meetings may provide clarification of the proposed approaches, including monitoring and data verification. SAEs require construction by the investigators of an accompanying narrative which may require detail beyond that collected by the research team access routinely-collected EHR source, but which should be available to the responsible investigator.

Methodological engagement, such as from MRC-NIHR Trials Methodology Research Partnership[46] will help to assess the settings for which using routinely-collected data are, or are not, more suitable than trial-specific data collection. A Study Within A Trial (SWAT) approach[47-49] can efficiently assess particular elements of EHR trials, such as from the WOSCOPS trial.[50,51] Collation of findings from retrospective comparisons will elucidate issues for prospective clarification and highlight challenges to address around better data collection.[26]

The impact of 2020's SARS-COV-2 pandemic was keenly felt by clinical trials with many hindered by a necessary pause, postponement or termination of recruitment and/or treatment. Routine hospital appointments became virtual and many research assessments had to follow. While trials teams could pivot to collect data online directly from participants, postponed or cancelled scans and tests will cause analysis and interpretation issues through the trial literature for many years which will need to be reported systematically using the CONSERVE extension to CONSORT currently in development. National strategic prioritisation of trials opened doors to routinely-collected data with uncommon rapidity. This

For peer review only - http://bmjopen.bmj.com/site/about/guidelines.xhtml

may allow the development of new access routes to data for trials and more wide-spread adoption by the research community of EHR-based methodology within trial delivery.

The UK has led by example in patients and public involvement and engagement (PPIE) for trials,[52,53] and key funding bodies support clear PPI. Patient ambassadors may be particularly helpful in informing the development of data-enabled trials. Locating participants via their EHR will enable participants to be informed of the trial's results, which, surprisingly, still does not always happen, although there is limited information available on best practice to inform participants; the Show Respect study is addressing this.[54] The StatinWISE study, a non-randomised cohort N-of-1 study of stopping statins in patients who have had heart attacks presented each patient a booklet of their own results before the study reported overall findings.[55] There is a clear need to engage the public on clinical trials, sensitising communities to research, and with a specific focus on the capacity of EHR to provide better outcomes.

This workshop showcased key successes and high-profile case studies (**Boxes 3-7**) that have answered questions where EHR data could map well to the trial questions, supplemented by some trial-specific collection. Over-interpretation of the clinical trials regulations can be risk-averse and suffocate innovation. Simplicity in implementation need not be interpreted as basic in ambition: straightforward protocols can still address a number of key clinical questions simultaneously.[1,56] Knowledge sharing and capability-building exercises now should facilitate future data-enabled trials. Trialists are getting better at writing up their findings and, increasingly, their designs, but further effort is required in writing up their practical implementation experiences. Best practice and case studies, reflecting on successes and challenges, should be shared, including in the threaded publications from NIHR HTA monographs[57] or a new, dedicated national forum.

Five years previously, NIHR launched a call for trials using routinely collected data which elicited a modest response form research community. Stimulating research using routinely collected clinical data has

For peer review only - http://bmjopen.bmj.com/site/about/guidelines.xhtml

remained a sustained interest of NIHR and launching out from this workshop, NIHR announced a new call for data-enabled trials.[4] NIHR committees reviewed 18 applications and the funded trials were confirmed in 2020 (**Table-1**).

Routinely-collected EHR offer great potential for faster, more efficient and less expensive trials reaching a broader catchment of the UK population. Wider adoption of EHR-based approaches and experiences from these studies, such as those funded from the NIHR data-enabled trial call, will provide the evidence to assess where EHR enablement brings benefits to trial execution and follow-up. Key recommendations from the workshop are presented in **Table_2**, many of which are now being addressed. Using EHR data for trials can be done and could be done at a greater scale than is the current practice. The desire to access and efficiently use these data is widely discussed and this workshop demonstrated that this aspiration is possible. The opportunities to learn and harness the power of information and technology to improve healthcare outcomes for everyone has never been better.

For peer review only

For peer review only - http://bmjopen.bmj.com/site/about/guidelines.xhtml

BOXES, TABLES AND FIGURES

Box-1: What are routinely-collected electronic health records?

There are vast amounts of data routinely being collected on patients, for example, in patient electronic health records (EHR), as well as disease and patient registries. Such data is commonly called real-world data (RWD), reflecting that it is routinely collected while patients go about their regular lives, as opposed to being specifically collected in a clinical trial. Because the NHS is a universal healthcare provider, free at point of care, the NHS EHR offers the possibility to collect the longitudinal medical history of 98% of the UK population who uses the NHS. EHR therefore provide an excellent research tool for studying population health. Primary care EHR are recorded by GPs and contain longitudinal demographic and clinical patient information encompassing symptoms, diagnoses, prescribing, laboratory tests and data from hospital visits and admissions. Primary care EHR can be collected and made available for research in near real-time. Secondary care records capture interactions that take place in a specific hospital and are event based, not longitudinal. Secondary care records are coded for reimbursement purposes, and not usually by clinicians. There is a time lag between patient hospital visits and secondary care EHR data being made available for research and NHS planning purposes.

For peer review only

For peer review only - http://bmjopen.bmj.com/site/about/guidelines.xhtml

Box-2: NIHR, HDR UK and CPRD

National Institute for Health Research

NIHR, a virtual organisation, is the research arm of the NHS. It comprises an Academy of trainees and senior investigators, and systems to make research processing easier and faster, including infrastructure, such as the clinical research network that plays a vital role in delivering research, and a number of research funding programmes. These include, for example, the NIHR Health Technology Assessment Programme, NIHR Health Services and Delivery Research and the NIHR Public Health Programme that fund prioritised health research for the public on behalf of the Department of Health and Social Care.

Health Data Research UK

HDR UK is the national institute for health data science. The institute, was established in 2018 as a joint strategic investment[1] with an aim to "unite the UK's health data to make discoveries that improve people's lives". Through initially six collaborative research sites across the UK and expanded to eight sites and 7 specialist HDR Hubs, the Health Data Research Innovation Gateway and Health Data Research Alliance, the Institute develops advanced health data science tools and technologies to analyse large-scale and diverse health data to solve complex health challenges. One priority themes is "Better, faster, and more efficient clinical trials".

Clinical Practice Research Datalink

CPRD is jointly sponsored by the Medicines and Healthcare products Regulatory Agency (MHRA) and NIHR, and is part of the Department of Health and Social Care. CPRD is the UK Government's dedicated real-world research service, which collects de-identified patient data on a daily basis from a network of one in every five GP practices across the UK, encompassing 25% of the UK population. The CPRD database includes longitudinal primary care records on a total 60 million patients collected over CPRD's 30 year history, with a median patient follow-up time of 10 years. Approximately 40% of CPRD's GP practice network are research-active.[16]

[1] Health Data Research UK is funded by the Medical Research Council, the health research departments of England, Scotland, Wales and Northern Ireland (National Institute for Health Research (NIHR), Chief Scientist Office (CSO), Health and Care Research Wales, HSC Research and Development respectively), the Economic and Social Research Council (ESRC), the Engineering and Physical Sciences Research Council (EPSRC), the British Heart Foundation and Wellcome.

For peer review only - http://bmjopen.bmj.com/site/about/guidelines.xhtml

For peer review only

For peer review only - http://bmjopen.bmj.com/site/about/guidelines.xhtml

Box-3: Key points from COPE study

**Study name (registration)**	COPE (Characterisation of COPD Exacerbations using Environmental Exposure Modelling)[8]
**Sponsorship**	Academic – Imperial, MRC funded
**Clinical setting**	Chronic obstructive pulmonary in primary care, UK only: national
**Design**	Patient-consented longitudinal cohort study (non-randomised)
**Size**	N=130
**Key outcome measure**	COPD exacerbations
**Rationale for presentation**	Design and feasibility
**Key discussion points**	The aim of the COPE study was to develop a method for predicting COPD exacerbations as a result of environmental exposures. Quality algorithmic query specification to identify patient cohorts in EHR relies on data quality. The algorithm for COPE was validated in a previous study by comparing the EHR records with additional information provided by GPs.[58] The validated query was applied centrally to search for patients in the CPRD database. GPs were then contacted and asked to take part in confirming patient eligibility from a pre-screened patient list.<br><br>Centralised searching of the EHR greatly minimised GP screening time in confirming patient eligibility. It was also possible for a GP to exclude patients who may not be suitable to take part (e.g. due to caring for a sick relative). No algorithm could reliably account for these types of patient exclusions.<br><br>Lessons from the study were that it is feasible to recruit patients from EHR and readily collect data for long-term follow-up, although this method does depend on GP participation. Using EHR meant far less effort than manual screening of GP records. Because of the specificity of the EHR and clinical validation steps, more appropriate patients were approached which reduced time needed for patient screening. Patients approached were keen to be involved in the study and as a result the enrolment was higher than using conventional methods.

For peer review only

Box-4: Key points from DECIDE trial

**Trial name (registration)**	Pragmatic Randomised 104 Week Multicentre Trial to Evaluate the Comparative Effectiveness of dapagliflozin and Standard of Care in Type-2 Diabetes. The DECIDE Study[17]
**Sponsorship**	Industry then academic -- AstraZeneca then Liverpool
**Clinical setting**	Type-2 diabetes mellitus on metformin and needing better glucose control in primary care setting, UK only: national
**Design**	Phase 4, randomised, open label, comparator trial
**Size**	N>800 (ongoing)
**Key outcome measure**	Clinical success (glucose control) at 12 months
**Rationale for presentation**	Recruitment
**Key discussion points**	DECIDE is a clinical effectiveness trial comparing dapagliflozin, a SGLT2 inhibitor, to standard care (alternative medications such as sulfonylureas and DPP4 inhibitors) in people with Type-2 diabetes requiring second-line pharmacological treatment (after metformin). There was a limited window in which patients need to be approached before treatment is changed.<br>CPRD managed the trial using their interventional research services platform which supported GPs and patients logging into the system. Algorithms were developed to carry out daily searches of primary care EHR for potentially eligible patients meeting pre-specified criteria. The primary care team then reviewed the search list and enrol eligible patients into the study at their next routine scheduled visit.<br>Most of the data required for the trial was available in CPRD's routinely-collected dataset, thereby facilitating collection of primary and secondary outcome measures as well as identification and recruitment. Patient-reported outcome measures were collected electronically. Trial monitoring could therefore be carried out centrally.<br>Lessons from the trial are that EHR search algorithms are easy to set up but feasibility assessments should take account of variability of coding and real-world clinical practice. Recruiting patients via EHR is achievable and allows easy collection of long-term data for follow-up but as with all trials it depends on GP and patient participation and engagement. Data-enablement and platform technology allows real-time adaptation to real-time conditions.

For peer review only

For peer review only - http://bmjopen.bmj.com/site/about/guidelines.xhtml

Box-5: Key points from ASCEND trial

**Trial name (registration)**	ASCEND[19,20]
**Sponsorship**	Academic – Oxford
**Clinical setting**	Diabetes mellitus, UK only: national, mail-based methods – no study sites
**Design**	2 x 2 factorial design randomised placebo-controlled trial (Phase 4)
**Size**	N=15000
**Key outcome measure**	Cardiovascular events
**Rationale for presentation**	Recruitment, conduct and follow-up
**Key discussion points**	ASCEND used highly streamlined mail-based methods to identify, recruit and follow-up (for an average of 7.5 years) 15,500 UK patients, making it one of the longest-duration and largest ever trials in diabetes.<br>Participants completed 6-monthly follow-up questionnaires, initially using paper and paper but later moving to an online system for those who wished.<br>The trialists gained access to HES data during the closing stages of the study, allowing additional follow-up information to be gained for those who had not returned a recent questionnaire. Subsequent analyses of the HES data compared with questionnaire data showed good concordance, suggesting that, in the UK, HES data alone could be used as a highly efficient means of follow-up for cardiovascular trials in the future.

For peer review only

For peer review only - http://bmjopen.bmj.com/site/about/guidelines.xhtml

Box-6: Key points from Salford Lung Study

**Trial name (registration)**	Salford Lung Study[21]
**Sponsorship**	Industry – GlaxoSmithKline
**Clinical setting**	Chronic obstructive pulmonary, UK only: regional
**Design**	Early-phase, cluster randomised trial run in one geographic location
**Size**	N=7200
**Key outcome measure**	COPD exacerbation rates
**Rationale for presentation**	Conduct and follow-up, collecting data on benefits and harms
**Key discussion points**	The study was the first in the world to have evaluated the effectiveness and safety of a pre-licenced medicine compared with standard of care when used in every day clinical practice in patients with COPD. Patients were randomised at their routine GP respiratory review visit and constant monitored through the 12 month study via real time data collection from GP and hospitals. Data was also collected from participating community pharmacies and from ONS mortality and Secondary User Service national datasets and an out of hours phone service. A safety alerting and reporting system was established based on serious adverse events being initially flagged in the EHR followed by review by the safety team and the principal investigator prior to submission to the sponsor as appropriate.<br>Lessons learnt were to ensure at the design phase that all the safety and endpoints of the study could be readily captured and the importance of early engagement with the regulators and NICE. The study demonstrated that safety monitoring in this type of real wold study was effective, could be done close to real time and was highly configurable. Widespread adoption could improve safety and such systems lend themselves to novel trial designs.

For peer review only

For peer review only - http://bmjopen.bmj.com/site/about/guidelines.xhtml

Box-7: Key points from SANAD-II trial

**Trial name (registration)**	SANAD-II[28,29]
**Sponsorship**	Academic – Liverpool
**Clinical setting**	Epilepsy
**Design**	Randomised controlled trial
**Size**	N=1510
**Key outcome measure**	Time to 12 months without seizure
**Rationale for presentation**	Analysis and interpretation
**Key discussion points**	The SANAD-II (Standard and New Antiepileptic Drugs) trial. SANAD II is a pragmatic, UK, multicentre, Phase IV RCT funded by the National Institute for Health Research (NIHR) Health Technology Assessment (HTA) programme, assessing the clinical and cost-effectiveness of a number of antiepileptic drugs as first-line treatments for newly diagnosed epilepsy. The primary outcome measure related to a period without seizures. These data were recorded on Case Report Forms by the treating clinical team at outpatient visits.<br><br>In a study of 98 patients, the team found the seizures were poorly recorded in EHR. Using only EHR data estimated that 74% of patients had spent one year seizure-free, whereas using only CRF data estimated that only 46% of patients had spent one year seizure-free. This is a clinically important difference. Researchers need to be able to trust in the completeness of EHR data to use them in reliable analyses or to understand how these data might supplement CRFs. This example cautions against naïve use of routinely-collected EHR data.

For peer review only - http://bmjopen.bmj.com/site/about/guidelines.xhtml

Table-1: Trials funded in Summer-2020 by first round of NIHR Data-Enabled Trials Call

NIHR panel	NIHR Ref	Trial title	Chief Investigator (Co-Chief Investigator)	Trials Unit	Planned start
HTA	NIHR130508	**UK-ROX:** Evaluating the clinical and cost-effectiveness of a conservative approach to oxygen therapy for invasively ventilated adults in intensive care	Daniel Martin	The Intensive Care National Audit and Research Centre	Q3-2020
HTA	NIHR130280	**DaRe2THINK:** Preventing stroke, premature death and cognitive decline in a broader community of patients with atrial fibrillation using healthcare data for pragmatic research	Dipak Kotecha	Birmingham CTU	Q4-2020
HTA	NIHR130573	**VITALL** Volatile vs Total intravenous Anaesthesia for major non-cardiac surgery – a pragmatic randomised controlled trial	Joyce Yeung	Warwick Clinical Trials Unit	Q4-2020
HTA	NIHR130548	**ASYMPTOMATIC:** Assessing Symptom-driven versus Maintenance Preventer Therapy for the Outpatient Management of Asthma In Children	Ian Sinha (Paula Willliamson)	Liverpool Clinical Trials Centre	Q1-2021
HS&DR	NIHR130075	In-silico trials of targeted screening for abdominal aortic aneurysm using linked healthcare data: can the efficiency of the NHS Abdominal Aortic Aneurysm Screening Programme be improved whilst maintaining publicly acceptable levels of disease detection in an ethically acceptable manner	Matthew Bown	n/a	Q4-2021
HS&DR	NIHR130581	Cluster randomised trial to improve antibiotic prescribing in primary care: individualised knowledge support during consultation for general practitioners and patients	Tjeerd van Staa	Manchester CTU	Q3-2020
HS&DR	NIHR130107	**ATHENA-M:** Observational study of age, test threshold and frequency on English national mammography screening outcomes	Sian Taylor-Phillips	n/a	Q4-2020

Key: HTA = Health Technology Assessment, HS&DR = Health Services and Delivery Research, PHR = Public Health Research
Note: 18 applications were submitted to the NIHR data enabled trials call across 3 research committees. 4 of 9 applications to HTA accepted for funding; 2 of 5 applications to HS&DR accepted for funding with 1 additional application still to be decided; 0 of 3 application to Public Health Research accepted for funding

For peer review only - http://bmjopen.bmj.com/site/about/guidelines.xhtml

Table-2: Recommendations for data-enabled trials

- Wider adoption of data-enabled methods for clinical trials
- Build in methodology questions into the trial protocol eg conversion rates for identification to recruitment; refining the process for identifying potential patients; understanding event rates for the target population
- Early engagement of regulators with regards to monitoring and data verification
- Patients and public involvement to robustly inform data-enabled trials
- Increased inter-operability of EHR systems
- Clear map for access to EHR: what is captured where, what do the data mean and how can they be used
- Consistency between core outcome sets used for research and routine practice
- Wider sharing and reporting of EHR codelists to support recruitment and outcome measures
- Sharing of detailed examples of success stories
- Capacity- and capability-building for future data-enabled trials

For peer review only

For peer review only - http://bmjopen.bmj.com/site/about/guidelines.xhtml

REFERENCES

1. Recovery Collaborative Group, Horby P, Lim WS, et al. Dexamethasone in Hospitalized Patients with Covid-19 - Preliminary Report. *N Engl J Med* 2020.

2. Office for Life Sciences. Life sciences: industrial strategy. 2017. https://www.gov.uk/government/publications/life-sciences-industrial-strategy (accessed 2020-11-04.

3. NHS Digital. Transforming health and care through technology. 2020. https://digital.nhs.uk/about-nhs-digital/our-work/transforming-health-and-care-through-technology (accessed 2020-08-11.

4. National Institute of Health Research (NIHR). NIHR call for data-enabled trials. 2019. https://www.nihr.ac.uk/funding-and-support/funding-opportunities/data-enabled-trials/9417.

5. Clinical Practice Research Datalink. Interventional Studies. 2020. https://www.cprd.com/interventional-studies (accessed 2020-09-12.

6. NHS Digital, HDRUK DigiTrials Hub. https://digital.nhs.uk/services/nhs-digitrials (accessed 2020-07-09.

7. Heatlh Data Research UK. Pioneering data research centres to enable cutting-edge research and innovation to benefit UK patients. 2019. https://www.hdruk.ac.uk/news/pioneering-data-research-centres-to-enable-cutting-edge-research-and-innovation/ (accessed 2020-08-11.

8. Quint JK, Moore E, Lewis A, et al. Recruitment of patients with Chronic Obstructive Pulmonary Disease (COPD) from the Clinical Practice Research Datalink (CPRD) for research. *NPJ Prim Care Respir Med* 2018; **28**(1): 21.

9. Walters SJ, Bonacho Dos Anjos Henriques-Cadby I, Bortolami O, et al. Recruitment and retention of participants in randomised controlled trials: a review of trials funded and published by the United Kingdom Health Technology Assessment Programme. *BMJ open* 2017; **7**(3): e015276.

10. National Institute of Health Research (NIHR). Primary Care Specialty Profile: Why you should deliver primary care research in the UK. 2020. https://www.nihr.ac.uk/documents/primary-care-specialty-profile/11603 (accessed 2020-09-12.

11. Gulliford MC, Prevost AT, Charlton J, et al. Effectiveness and safety of electronically delivered prescribing feedback and decision support on antibiotic use for respiratory illness in primary care: REDUCE cluster randomised trial. *BMJ* 2019; **364**: l236.

12. Gulliford MC, Juszczyk D, Prevost AT, et al. Electronically delivered interventions to reduce antibiotic prescribing for respiratory infections in primary care: cluster RCT using electronic health records and cohort study. *Health technology assessment* 2019; **23**(11): 1-70.

13. NHS Scotland. SHARE. https://www.registerforshare.org/ (accessed 2019-07-03.

14. NHS. Coronavirus vaccine research. 2020. https://www.nhs.uk/conditions/coronavirus-covid-19/research/coronavirus-vaccine-research/ (accessed 2020-10-23.

15. Di Angelantonio E, Thompson SG, Kaptoge S, et al. Efficiency and safety of varying the frequency of whole blood donation (INTERVAL): a randomised trial of 45 000 donors. *The Lancet* 2017; **390**(10110): 2360-71.

16. Lai YS, Afseth JD. A review of the impact of utilising electronic medical records for clinical research recruitment. *Clin Trials* 2019; **16**(2): 194-203.

For peer review only - http://bmjopen.bmj.com/site/about/guidelines.xhtml

17. Murphy ME, Byrne M, Boland F, et al. Supporting general practitioner-based care for poorly controlled Type-2 diabetes mellitus (the DECIDE study): feasibility study and protocol for a pilot cluster randomised controlled trial. *Pilot Feasibility Stud* 2018; **4**: 159.

18. clinicaltrials.gov. A Randomized Trial Assessing the Effects of Inclisiran on Clinical Outcomes Among People With Cardiovascular Disease (ORION-4). 2018. https://clinicaltrials.gov/ct2/show/NCT03705234 (accessed 2020-08-11.

19. Ascend Study Collaborative Group, Bowman L, Mafham M, et al. Effects of Aspirin for Primary Prevention in Persons with Diabetes Mellitus. *N Engl J Med* 2018; **379**(16): 1529-39.

20. Ascend Study Collaborative Group. ASCEND: A Study of Cardiovascular Events iN Diabetes. https://clinicaltrials.gov/ct2/show/NCT00135226 (accessed 2019-07-03.

21. Vestbo J, Leather D, Diar Bakerly N, et al. Effectiveness of Fluticasone Furoate-Vilanterol for COPD in Clinical Practice. *N Engl J Med* 2016; **375**(13): 1253-60.

22. Woodcock A, Vestbo J, Bakerly ND, et al. Effectiveness of fluticasone furoate plus vilanterol on asthma control in clinical practice: an open-label, parallel group, randomised controlled trial. *The Lancet* 2017; **390**(10109): 2247-55.

23. World Health Organisation. Specifications for Pharmaceutical Preparations -- Annex 5: Guidance on good data and record management practices. 2014. https://www.who.int/medicines/publications/pharmprep/WHO_TRS_996_annex05.pdf (accessed 2020-10-23.

24. Public Health England. Simulacrum: artificial patient-like cancer data to help researchers gain insights. https://healthdatainsight.org.uk/project/the-simulacrum/ (accessed 2019-07-03.

25. Health Data Research Innovation Gateway. Health Data Research Innovation Gateway: Explore datasets, tools and resources used in health research across the UK. 2020. https://www.healthdatagateway.org/ (accessed 2020-08-11.

26. Ahmed S, Sydes M, Love S, Carpenter J. Assessing the agreement of routinely-collected health data and case record form data in randomised controlled trials: a systematic review (CRD42020186048). 2020. https://www.crd.york.ac.uk/prospero/display_record.php?ID=CRD42020186048.

27. Sydes MR, Love SB, Murray ML, Lensen S, Macnair A, Carpenter J. SWAT 125: Comparison of trial-collected and routinely-collected death data. Queens University Belfast, 2020

28. Powell GA, Bonnett LJ, Tudur-Smith C, Hughes DA, Williamson PR, Marson AG. Using routinely recorded data in the UK to assess outcomes in a randomised controlled trial: The Trials of Access. *Trials* 2017; **18**(1): 389.

29. University of Liverpool, National Institute of Health Research (NIHR). Trial comparing the effectiveness and cost effectiveness of levetiracetam and zonisamide versus standard treatments for epilepsy: a comparison of Standard And New Antiepileptic Drugs (ISRCTN30294119). http://www.sanad2.org.uk/ (accessed 2019-07-03.

30. Pate A, Barrowman M, Webb D, et al. Study investigating the generalisability of a COPD trial based in primary care (Salford Lung Study) and the presence of a Hawthorne effect. *BMJ Open Respir Res* 2018; **5**(1): e000339.

31. Lensen S, Macnair A, Love SB, et al. Access to routinely collected health data for clinical trials - review of successful data requests to UK registries. *Trials* 2020; **21**(1): 398.

32. McKay AJ, Jones AP, Gamble CL, Farmer AJ, Williamson PR. Use of routinely collected data in a UK cohort of publicly funded randomised clinical trials. *F1000Research* 2020; **9**(323): 323.

For peer review only - http://bmjopen.bmj.com/site/about/guidelines.xhtml

33. NHS England. Data quality improvement. https://www.england.nhs.uk/data-services/validate/ (accessed 2020-08-11.

34. UK Health Data Research Alliance. UK Health Data Research Alliance. https://www.hdruk.ac.uk/digital-innovation-hubs/uk-health-data-research-alliance/ (accessed 2019-07-14.

35. Paprica PA, Sydes M, McGrail K, Morris A, Schull MJ, Walker R. Prospective data linkage to facilitate COVID-19 trials – A call to action. *International Journal of Population Data Science* 2020; **5**(2).

36. Chalmers I, Glasziou P. Avoidable waste in the production and reporting of research evidence. *Lancet* 2009; **374**(9683): 86-9.

37. Djulbegovic B, Kumar A, Glasziou P, Miladinovic B, Chalmers I. Medical research: Trial unpredictability yields predictable therapy gains. *Nature* 2013; **500**(7463): 395-6.

38. Glasziou P, Chalmers I. Research waste is still a scandal-an essay by Paul Glasziou and Iain Chalmers. *BMJ* 2018; **363**: k4645.

39. Department of Health and Social Care, Hancock MP M. NHS recovery to put staff wellbeing at its heart. 2020. https://www.gov.uk/government/news/nhs-recovery-to-put-staff-wellbeing-at-its-heart (accessed 2020-08-11.

40. Fogel DB. Factors associated with clinical trials that fail and opportunities for improving the likelihood of success: A review. *Contemp Clin Trials Commun* 2018; **11**: 156-64.

41. Getz KA, Zuckerman R, Cropp AB, Hindle AL, Krauss R, Kaitin KI. Measuring the Incidence, Causes, and Repercussions of Protocol Amendments. *Drug Information Journal* 2011; **45**(3): 265-75.

42. Clark LT, Watkins L, Pina IL, et al. Increasing Diversity in Clinical Trials: Overcoming Critical Barriers. *Curr Probl Cardiol* 2019; **44**(5): 148-72.

43. Marum RJ. Underrepresentation of the elderly in clinical trials, time for action. *British Journal of Clinical Pharmacology* 2020; **86**(10): 2014-6.

44. Bower P, Grigoroglou C, Anselmi L, et al. Is health research undertaken where the burden of disease is greatest? Observational study of geographical inequalities in recruitment to research in England 2013-2018. *BMC Med* 2020; **18**(1): 133.

45. National Institute of Health Research. The Include Ethnicity Framework. 2020. https://www.trialforge.org/trial-forge-centre/include/ (accessed 2020-11-19.

46. Medical Research Council (MRC), National INstitute of Health Research (NIHR). MRC-NIHR Trials Methodology Research Partnership (TMRP). 2019. https://www.methodologyhubs.mrc.ac.uk/about/tmrp/ (accessed 2019-07-03.

47. Treweek S, Bevan S, Bower P, et al. Trial Forge Guidance 1: what is a Study Within A Trial (SWAT)? *Trials* 2018; **19**(1): 139.

48. Treweek S. Why the NIHR's new funding stream for 'Studies Within A Trial (SWATs) is potentially game-changing. 2018.

49. MRC Hubs for Trials Methodology Research. SWAT Repository Store https://www.qub.ac.uk/sites/TheNorthernIrelandNetworkforTrialsMethodologyResearch/SWATSWARInformation/Repositories/SWATStore/ (accessed 2020-09-13.

For peer review only - http://bmjopen.bmj.com/site/about/guidelines.xhtml

50. Barry SJ, Dinnett E, Kean S, Gaw A, Ford I. Are routinely collected NHS administrative records suitable for endpoint identification in clinical trials? Evidence from the West of Scotland Coronary Prevention Study. *PLoS One* 2013; **8**(9): e75379.

51. The West of Scotland Coronary Prevention Study Group. Computerised record linkage: compared with traditional patient follow-up methods in clinical trials and illustrated in a prospective epidemiological study. *J Clin Epidemiol* 1995; **48**(12): 1441-52.

52. South A, Bailey J, Parmar MKB, Vale CL. Effectiveness and acceptability of methods of communicating the results of clinical research to lay and professional audiences: protocol for a systematic review. *Syst Rev* 2019; **8**(1): 150.

53. South A, Hanley B, Gafos M, et al. Models and impact of patient and public involvement in studies carried out by the Medical Research Council Clinical Trials Unit at University College London: findings from ten case studies. *Trials* 2016; **17**: 376.

54. South A, UCL MCTUa. Show results to participants engaged in clinical trials. 2019. http://www.isrctn.com/ISRCTN96189403.

55. Herrett E, Williamson E, Beaumont D, et al. Study protocol for statin web-based investigation of side effects (StatinWISE): a series of randomised controlled N-of-1 trials comparing atorvastatin and placebo in UK primary care. *BMJ open* 2017; **7**(12): e016604.

56. Parmar MK, Barthel FM, Sydes M, et al. Speeding up the evaluation of new agents in cancer. *J Natl Cancer Inst* 2008; **100**(17): 1204-14.

57. National Institute of Health Research (NIHR). NIHR Journals Library: Information for Authors. 2019.

58. Rothnie KJ, Mullerova H, Hurst JR, et al. Validation of the Recording of Acute Exacerbations of COPD in UK Primary Care Electronic Healthcare Records. *PLoS One* 2016; **11**(3): e0151357.

For peer review only

For peer review only - http://bmjopen.bmj.com/site/about/guidelines.xhtml

AUTHOR LIST

Name	ORCID	Affiliation
Sydes, Matthew	0000-0002-9323-1371	MRC Clinical Trials Unit at UCL, Institute of Clinical Trials and Methodology, UCL, London, UK & HDR UK
Barbachano, Yolanda	none	Medicines and Healthcare products Regulatory Agency (MHRA)
Bowman, Louise	0000-0003-1125-8616	Clinical Trial Service Unit and Epidemiological Studies Unit, Nuffield Department of Population Health, University of Oxford, Oxford
Denwood, Tom	0000-0002-7337-2425	NHS Digital
Farmer, Andrew	0000-0002-6170-4402	Nuffield Department of Primary Health Care Sciences, University of Oxford.
Garfield-Birkbeck, Steph	0000-0002-3181-2840	National Institute for Health Research Evaluation, Trials and Studies Coordinating Centre
Gibson, Martin	0000-0002-1331-1524	NWEH Ltd
Gulliford, Martin	0000-0003-1898-9075	Kings College London, London
David Harrison	0000-0002-9002-9098	Intensive Care National Audit & Research Centre (ICNARC), London, UK
Hewitt, Catherine	0000-0002-0415-3536	York Trials Unit, Department of Health Sciences, University of York
Logue, Jennifer	0000-0001-9549-2738	Lancaster University
Navaie, Will	none	Health Research Authority
Norrie, John	0000-0001-9823-9252	Edinburgh Clinical Trials Unit, University of Edinburgh
O'Kane, Martin	none	Medicines and Healthcare products Regulatory Agency (MHRA)
Quint, Jennifer	0000-0003-0149-4869	NHLI, Imperial College London, UK
Rycroft-Malone, Jo	0000 0003 3858 5625	Lancaster University & NIHR Health Services & Delivery Programme
Sheffield, Jonathan	None	Formerly CEO NIHR Clinical Research Network; University of Leeds
Smeeth, Liam	0000-0002-9168-6022	London School of Hygiene and Tropical Medicine

For peer review only - http://bmjopen.bmj.com/site/about/guidelines.xhtml

Name	ORCID	Affiliation
Sullivan, Frank	0000-0002-6623-4964	Division of Population & Behavioural Science, University of St. Andrews<br>Department of Family & Community Medicine, University of Toronto
Tizzard, Juliet	None	Health Research Authority
Walker, Paula	None	Medicines and Healthcare products Regulatory Agency (MHRA)
Wilding, John	0000-0003-2839-8404	Department of Cardiovascular and Metabolic Medicine<br>Institute of Life Course and Medical Sciences<br>University of Liverpool
Williamson, Paula	0000-0001-9802-6636	University of Liverpool
ɸ Landray, Martin	0000-0001-6646-827X	• Nuffield Department of Population Health, University of Oxford, Oxford, UK<br>• Health Data Research UK, University of Oxford, Oxford, UK<br>• NIHR Oxford Biomedical Research Centre, Oxford University Hospitals NHS Foundation Trust, Oxford, UK
ɸ Morris, Andrew	0000-0002-1766-0473	Health Data Research UK
ɸ Walker, Rhoswyn	None	Health Data Research UK
ɸ Williams, Hwyel	0000-0002-5646-3093	Professor of Dermato-Epidemiology, University of Nottingham
ɸ Valentine, Janet	None	Clinical Practice Research Datalink
And the Data Enabled Trials Workshop Group (see member list)		

ɸ These authors contributed equally

For peer review only - http://bmjopen.bmj.com/site/about/guidelines.xhtml

The **Data Enabled Trials Group Workshop Group** members are:

Name	ORCID	Affiliation
Ashby, Deborah	TBC	Imperial College London
Barbachano, Yolanda	none	Medicines and Healthcare products Regulatory Agency (MHRA)
Beatty, Susan	TBC	CPRD
Bennie, Marion	TBC	HDR UK (Scotland)
Bodmer, Helen	TBC	Medical Research Council
Bowman, Louise	0000-0003-1125-8616	Clinical Trial Service Unit and Epidemiological Studies Unit, Nuffield Department of Population Health, University of Oxford, Oxford
Brady, Emer	TBC	Leicester General Hospital
Brocklehurst, Paul	TBC	North Wales Organisation for Randomised Trials
Brooke, Jackson	TBC	CPRD
Campbell, Marion	TBC	HDR UK (Scotland)
Cipriani, Andrea	TBC	University of Oxford
Daniels, Jane	TBC	University of Nottingham
Denwood, Tom	0000-0002-7337-2425	NHS Digital
Donnelly, Michael	TBC	Queen's University Belfast
Dreischulte, Tobias	TBC	University of Dundee
Edwards, Mark	TBC	University Hospital Southampton NHSFT
Farmer, Andrew	0000-0002-6170-4402	Nuffield Department of Primary Health Care Sciences, University of Oxford.
Freemantle, Nick	TBC	Comprehensive Clinical Trials Unit, UCL
Garfield-Birkbeck, Steph	0000-0002-3181-2840	National Institute for Health Research Evaluation, Trials and Studies Coordinating Centre
Gibson, Martin	0000-0002-1331-1524	NWEH Ltd
Goodacre, Steve	TBC	NIHR HTA CET
Gould, Doug	TBC	Intensive Care National Audit & Research Centre (ICNARC)
Gray, Laura	TBC	University of Leicester
Griffin, Xavier	TBC	HDR UK Oxford
Gulliford, Martin	0000-0003-1898-9075	Kings College London, London
Harrison, David	0000-0002-9002-9098	Intensive Care National Audit & Research Centre (ICNARC), London, UK
Hewitt, Catherine	0000-0002-0415-3536	York Trials Unit, Department of Health Sciences, University of York
Hiemstra, Thomas	TBC	University of Cambridge
Higgins, Anna	TBC	University of Nottingham
Hippisley-Cox, Julia	TBC	TBC
King, Michael	TBC	PRIMENT Clinical Trials Unit, UCL
Korniak, Sasha	TBC	Intensive Care National Audit & Research Centre (ICNARC)
Landray, Martin	0000-0001-6646-827X	Nuffield Department of Population Health, University of Oxford, Oxford, UK; Health Data Research UK, University of Oxford, Oxford, UK; NIHR Oxford Biomedical Research Centre, Oxford University Hospitals NHS Foundation Trust, Oxford, UK
Lewis, Martyn	TBC	Keele Clinical Trials Unit
Lobban, Fiona	TBC	Lancaster University
Logue, Jennifer	0000-0001-9549-2738	Lancaster University
Lopez Campos, Guillermo	TBC	Queen's University Belfast
MacNeill, Stephanie	TBC	University of Bristol
Mafham, Marion	TBC	HDR UK Oxford
McCowan, Colin	TBC	HDR UK Scotland
Meeley, Kathleen	TBC	(Previously) Medicines and Healthcare products Regulatory Agency (MHRA)
Michael, Agnieszka	TBC	Surrey Trials Unit
Mills, Nick	TBC	BHF Centre for Cardiovascular Sciences &
Morris, Andrew	0000-0002-1766-0473	Health Data Research UK

For peer review only - http://bmjopen.bmj.com/site/about/guidelines.xhtml

Name	ORCID	Affiliation
Navaie, Will	none	Health Research Authority
Nazareth, Irwin	TBC	PRIMENT Clinical Trials Unit, UCL
Newby, Chris	TBC	Queen Mary University of London
Norrie, John	0000-0001-9823-9252	Edinburgh Clinical Trials Unit, University of Edinburgh
Nuttall, Jacqui	TBC	Southampton Clinical Trials Unit
O'Kane, Martin	None	Medicines and Healthcare products Regulatory Agency (MHRA)
Quint, Jennifer	0000-0003-0149-4869	NHLI, Imperial College London, UK
Rai, Dheeraj	TBC	University of Bristol
Roberts, Ian	TBC	London School of Hygience & Tropical Medicine Clinical Trials Unit
Robling, Mike	TBC	University of Cardiff
Rycroft-Malone, Jo	0000 0003 3858 5625	Lancaster University & NIHR Health Services & Delivery Programme
Sheffield, Jonathan	None	Formerly CEO NIHR Clinical Research Network; University of Leeds
Sheikh, Aziz	TBC	HDR UK (Scotland)
Shyamsundar, Murali	TBC	Queen's University Belfast
Smeeth, Liam	0000-0002-9168-6022	London School of Hygiene and Tropical Medicine
Snowdon, Claire	TBC	ICR Clinical Trials & Statistics Unit (ICR-CTSU)
Soames, Jamie	TBC	NIHR
Stewart, Derek	TBC	PPI
Streeter, Adam	TBC	University of Plymouth
Sullivan, Frank	0000-0002-6623-4964	Division of Population & Behavioural Science, University of St. Andrews; Department of Family & Community Medicine, University of Toronto
Sydes, Matthew	0000-0002-9323-1371	MRC Clinical Trials Unit at UCL, Institute of Clinical Trials and Methodology, UCL, London, UK & HDR UK
Tembo, Doreen	TBC	NIHR Evaluation, Trials and Studies Coordinating Centre (NETSCC)
Tizzard, Juliet	None	Health Research Authority
Tudur Smith, Catrin	TBC	University of Liverpool
Valentine, Janet	TBC	Clinical Practice Research Datalink
Walker, Paula	None	Medicines and Healthcare products Regulatory Agency (MHRA)
Walker, Rhoswyn	None	Health Data Research UK
Watkins, Alan	TBC	University of Swansea
Whiteley, Will	0000-0003-2839-8404	Department of Cardiovascular and Metabolic Medicine; Institute of Life Course and Medical Sciences; University of Liverpool
Wilding, John	TBC	TBC
Williams, Hwyel	0000-0002-5646-3093	Professor of Dermato-Epidemiology, University of Nottingham
Williams, Kate	TBC	University of Cambridge
Williamson, Elizabeth	TBC	London School of HygienceHygiene & Tropical Medicine
Williamson, Paula	0000-0001-9802-6636	University of Liverpool

For peer review only - http://bmjopen.bmj.com/site/about/guidelines.xhtml

CONTRIBUTIONS

We will turn this in to text on editorial advice, depending on whether preferring grouping by author or grouping by contribution.

Name	Organised workshop	Attended workshop	Contributed to workshop discussion	Gave plenary presentation	Chaired session	Chaired discussion group	Member of discussion panel	Scribed workshop	Scribed discussion group	Led writing of manuscript	Wrote key sections of manuscript	Contributed to and agreed manuscript
**Named authors**												
Barbachano, Yolanda	.	X	X	.	.	.	X	.	.	.	.	X
Bowman, Louise	.	X	X	X	.	.	.	.	.	.	.	x
Denwood, Tom	.	X	X	X	.	.	.	.	.	.	.	X
Farmer, Andrew	.	X	X	.	X	.	.	.	.	.	.	X
Garfield-Birkbeck, Steph	.	X	X	.	.	.	.	X	.	.	.	X
Gibson, Martin	.	X	X	X	.	.	.	.	.	.	.	X
Gulliford, Martin	.	X	X	X	.	.	.	.	.	.	.	X
Harrison, David	.	X	X	.	.	X	.	.	X	.	.	X
Hewitt, Catherine	.	X	X	.	.	X	.	.	X	.	.	X
Landray, Martin	X	X	X	X	X	.	.	.	.	.	X	x
Logue, Jennifer	.	X	X	.	.	X	.	.	X	.	.	X
Morris, Andrew	X	X	X	.	X	.	.	.	.	.	.	x
Navaie, Will	.	X	X	.	.	.	X	.	.	.	.	X
Norrie, John	.	X	X	.	.	X	.	.	X	.	.	X
O'Kane, Martin	.	X	X	X	.	.	.	.	.	.	X	X
Quint, Jennifer	.	X	x	x	.	.	.	.	.	.	.	X
Rycroft-Malone, Jo	.	X	X	.	.	.	X	.	.	.	.	X
Sheffield, Jonathan	.	X	X	.	.	.	X	.	.	.	.	X

For peer review only - http://bmjopen.bmj.com/site/about/guidelines.xhtml

Name	Organised workshop	Attended workshop	Contributed to workshop discussion	Gave plenary presentation	Chaired session	Chaired discussion group	Member of discussion panel	Scribed workshop	Scribed discussion group	Led writing of manuscript	Wrote key sections of manuscript	Contributed to and agreed manuscript
Smeeth, Liam	.	X	X	X	.	.	.	.	.	.	.	X
Sullivan, Frank	.	X	X	.	.	X	.	.	X	.	.	X
Sydes, Matthew	.	X	X	.	.	.	.	X	.	X	X	x
Tizzard, Juliet	.	X	.	.	.	.	X	.	.	.	.	.
Valentine, Janet	X	X	X	X	.	.	.	.	.	X	X	x
Walker, Paula	.	(Apols)	.	.	.	.	.	.	.	.	X	x
Walker, Rhoswyn	X	X	X	.	.	.	.	.	.	.	x	X
Wilding, John	.	X	X	X	.	.	.	.	.	.	.	X
Williams, Hwyel	X	X	X	.	.	.	.	.	.	.	.	X
Williamson, Paula	.	X	X	X	.	X	.	.	.	.	X	X

For peer review only

COMPETING INTERESTS

J Wilding reports grants, personal fees and other from AstraZeneca, other from Astellas, other from Boehringer Ingelheim, other from Lilly, other from Napp, grants, personal fees and other from Novo Nordisk, other from Sanofi, grants, personal fees and other from Takeda, other from Rhythm Pharma, outside the submitted work.

The other authors do not declare relevant competing interests for this work.

For peer review only

For peer review only - http://bmjopen.bmj.com/site/about/guidelines.xhtml

BMJ Open

Realising the full potential of data-enabled trials in the UK: a call for action

Journal:	*BMJ Open*
Manuscript ID	bmjopen-2020-043906.R2
Article Type:	Communication
Date Submitted by the Author:	26-Apr-2021

Complete List of Authors:	Sydes, Matthew; Medical Research Council Clinical Trials Unit at University College London, MRC Clinical Trials Unit, Institute of Clinical Trials and Methodology<br>Barbachano, Yolanda; MHRA<br>Bowman, Louise; MRC Clinical Trial Service Unit and Epidemiological Studies Unit<br>Denwood, Tom; NHS Digital<br>Farmer, Andrew; University of Oxford, Nuffield Department of Primary Care Health Sciencesd<br>Garfield-Birkbeck, Steph; National Institute for Health Research Evaluation, Trials and Studies Coordinating Centre<br>Gibson, Martin; NWEH<br>Gulliford, Martin; King's College London, UK,<br>Harrison, David; ICNARC,<br>Hewitt, Catherine; The University of York, Health Sciences<br>Logue, Jennifer; Lancaster University<br>Navaie, Will; Health Research Authority<br>Norrie, John; Edinburgh Clinical Trials Unit, University of Edinburgh No. 9, Bioquarter<br>O'Kane, Martin; Medicines and Healthcare Products Regulatory Agency<br>Quint, Jennifer; Imperial College London, Respiratory Epidemiology, Occupational Medicine and Public Health<br>Rycroft-Malone, Jo; Lancaster University; NIHR Health Services & Delivery Programme<br>Sheffield, Jonathan; Formerly CEO NIHR Clinical Research Network; University of Leeds<br>Smeeth, Liam; London School of Hygiene and Tropical Medicine, Epidemiology and Population Health<br>Sullivan, Frank; University of St. Andrews, Division of Population & Behavioural Science; University of Toronto, Department of Family & Community Medicine<br>Tizzard, Juliet; Health Research Authority<br>Walker, Paula; Medicines and Healthcare Products Regulatory Agency<br>Wilding, John; University of Liverpool, School of Clinical Sciences<br>Williamson, Paula; University of Liverpool,<br>Landray, Martin; University of Oxford Nuffield Department of Population Health; University of Oxford Big Data Institute<br>Morris, Andrew; Health Data Research UK<br>Walker, Rhoswyn; Health Data Research UK<br>Williams, HC; University of Nottingham,<br>Valentine, Janet; Medicines and Healthcare Products Regulatory Agency,

	Clinical Practice Research Datalink<br>Data Enabled Trials Workshop Group, And the ; (see member list)
**Primary Subject Heading**:	Health informatics
Secondary Subject Heading:	Evidence based practice, Research methods
Keywords:	Health informatics < BIOTECHNOLOGY & BIOINFORMATICS, STATISTICS & RESEARCH METHODS, Clinical trials < THERAPEUTICS

SCHOLARONE™
Manuscripts

I, the Submitting Author has the right to grant and does grant on behalf of all authors of the Work (as defined in the below author licence), an exclusive licence and/or a non-exclusive licence for contributions from authors who are: i) UK Crown employees; ii) where BMJ has agreed a CC-BY licence shall apply, and/or iii) in accordance with the terms applicable for US Federal Government officers or employees acting as part of their official duties; on a worldwide, perpetual, irrevocable, royalty-free basis to BMJ Publishing Group Ltd ("BMJ") its licensees and where the relevant Journal is co-owned by BMJ to the co-owners of the Journal, to publish the Work in BMJ Open and any other BMJ products and to exploit all rights, as set out in our licence.

The Submitting Author accepts and understands that any supply made under these terms is made by BMJ to the Submitting Author unless you are acting as an employee on behalf of your employer or a postgraduate student of an affiliated institution which is paying any applicable article publishing charge ("APC") for Open Access articles. Where the Submitting Author wishes to make the Work available on an Open Access basis (and intends to pay the relevant APC), the terms of reuse of such Open Access shall be governed by a Creative Commons licence – details of these licences and which Creative Commons licence will apply to this Work are set out in our licence referred to above.

Other than as permitted in any relevant BMJ Author's Self Archiving Policies, I confirm this Work has not been accepted for publication elsewhere, is not being considered for publication elsewhere and does not duplicate material already published. I confirm all authors consent to publication of this Work and authorise the granting of this licence.

For peer review only

**Title:**	**Realising the full potential of data-enabled trials in the UK: a call for action**
**Running title:**	Data enabled trials in the UK
**Version and date:**	Version 2.01; (21-Apr-2021) -- Response to reviewers & editorial comments
**Tables and Figures:**	2 Tables<br>7 Boxes<br>0 Figures<br>0 Supplementary tables<br>0 Supplementary figures<br>0 Appendices

Authors:

Sydes, Matthew (0000-0002-9323-1371)
MRC Clinical Trials Unit at UCL, Institute of Clinical Trials and Methodology, UCL, London, UK
& HDR UK London

Barbachano, Yolanda
Medicines and Healthcare products Regulatory Agency (MHRA)

Bowman, Louise (0000-0003-1125-8616)
Clinical Trial Service Unit and Epidemiological Studies Unit, Nuffield Department of Population Health, University of Oxford, Oxford

Denwood, Tom (0000-0002-7337-2425)
NHS Digital

Farmer, Andrew (0000-0002-6170-4402)
Nuffield Department of Primary Health Care Sciences, University of Oxford.

Garfield-Birkbeck, Steph (0000-0002-3181-2840)
National Institute for Health Research Evaluation, Trials and Studies Coordinating Centre

Gibson, Martin (0000-0002-1331-1524)
NWEH Ltd

Gulliford, Martin (0000-0003-1898-9075)
Kings College London, London

David Harrison (0000-0002-9002-9098)
Intensive Care National Audit & Research Centre (ICNARC), London, UK

Hewitt, Catherine (0000-0002-0415-3536)
York Trials Unit, Department of Health Sciences, University of York

Logue, Jennifer (0000-0001-9549-2738)
Lancaster University

For peer review only - http://bmjopen.bmj.com/site/about/guidelines.xhtml

Navaie, Will
Health Research Authority

Norrie, John (0000-0001-9823-9252)
Edinburgh Clinical Trials Unit, University of Edinburgh

O'Kane, Martin
Medicines and Healthcare products Regulatory Agency (MHRA)

Quint, Jennifer (0000-0003-0149-4869)
NHLI, Imperial College London, UK

Rycroft-Malone, Jo (0000 0003 3858 5625)
Lancaster University & NIHR Health Services & Delivery Programme

Sheffield, Jonathan
Formerly CEO NIHR Clinical Research Network; University of Leeds

Smeeth, Liam (0000-0002-9168-6022)
London School of Hygiene and Tropical Medicine

Sullivan, Frank (0000-0002-6623-4964)
Division of Population & Behavioural Science, University of St. Andrews
Department of Family & Community Medicine, University of Toronto

Tizzard, Juliet
Health Research Authority

Walker, Paula
Medicines and Healthcare products Regulatory Agency (MHRA)

Wilding, John (0000-0003-2839-8404)
Department of Cardiovascular and Metabolic Medicine
Institute of Life Course and Medical Sciences
University of Liverpool

Williamson, Paula (0000-0001-9802-6636)
University of Liverpool

ᶲ Landray, Martin (0000-0001-6646-827X)
Nuffield Department of Population Health, University of Oxford, Oxford, UK
Health Data Research UK, University of Oxford, Oxford, UK
NIHR Oxford Biomedical Research Centre, Oxford University Hospitals NHS Foundation Trust, Oxford, UK

ᶲ Morris, Andrew (0000-0002-1766-0473)
Health Data Research UK

ᶲ Walker, Rhoswyn (0000-0001-5605-2879)
Health Data Research UK

For peer review only

For peer review only - http://bmjopen.bmj.com/site/about/guidelines.xhtml

ᶲ Williams, Hwyel (0000-0002-5646-3093)
Professor of Dermato-Epidemiology, University of Nottingham

ᶲ Valentine, Janet
Clinical Practice Research Datalink

*And the **Data Enabled Trials Workshop Group** (see notes)*

**Contributors:**	ᶲ These authors contributed equally<br><br>See list of author contributions at end of document
**Trial identification:**	N/A
**Correspondence:**	Matthew Sydes m.sydes@ucl.ac.uk
**Sponsor:**	N/A
**Funding &support:**	The workshop was co-funded by National Institute of Health Research (NIHR) Health Technology Assessment Programme, Clinical Practice Research Datalink (CPRD) and Health Data Research UK; MRC grant MC_UU_12023/24 funded the salary of Matthew Sydes
**Competing Interests**	All authors have completed the ICMJE uniform disclosure form at www.icmje.org/coi_disclosure.pdf. Please see the end of the manuscript for full list of author disclosures.
**Transparency declaration:**	All authors affirm that the manuscript is an honest, accurate, and transparent account of the study being reported; that no important aspects of the study have been omitted; and that any discrepancies from the study as planned (and, if relevant, registered) have been explained
**Ethical approval:**	(Not required)
**Data sharing:**	(No new data)

For peer review only

	Section	Words	Cumulative
**Word Count:**	Abstract	333	n/a
	Introduction	491	491
	Findings > Design	707	1198
	Findings > Recruitment	878	2076
	Findings > Conduct	587	2663
	Findings > Outcomes	831	3494
	Findings > Analysis	627	4121
	Discussion	1876	5997
	**Total**	**5997**	

(Word counts cover the body text of each section and exclude the headings and the ending notes)

For peer review only

For peer review only - http://bmjopen.bmj.com/site/about/guidelines.xhtml

ABSTRACT

Rationale: Clinical trials are the gold standard for testing interventions. COVID-19 has further raised their public profile, and emphasised the need to deliver better, faster, more efficient trials for patient benefit. Considerable overlap exists between data required for trials and data already collected routinely in electronic healthcare records (EHR). Opportunities exist to utilise these in innovative ways to decrease duplication of effort, and speed trial recruitment, conduct and follow-up.

Approach: The National Institute of Health Research (NIHR), Health Data Research UK (HDR UK) and Clinical Practice Research Datalink (CPRD) co-organised a national workshop to accelerate the agenda for "data-enabled clinical trials". Showcasing successful examples and imagining future possibilities, the plenary talks, panel discussions, group discussions and case studies covered: design/feasibility; recruitment; conduct/follow-up; collecting benefits/harms; and analysis/interpretation.

Reflection: Some notable studies have successfully accessed and used EHR to identify potential recruits, support randomised trials, deliver interventions and supplement/replace trial-specific follow-up. Some outcome measures are already reliably collected; others, like safety, need detailed work to meet regulatory reporting requirements. There is a clear need for system interoperability and a "route map" to identify and access the necessary datasets. Researchers running regulatory-facing trials must carefully consider how data quality and integrity would be assessed. An experience-sharing forum could stimulate wider adoption of EHR-based methods in trial design and execution.

Discussion: EHR offer opportunities to better plan clinical trials, assess patients and capture data more efficiently, reducing research waste and increasing focus on each trial's specific challenges. The short-term emphasis should be on facilitating patient recruitment and for post-marketing authorisation trials where research-relevant outcome measures are readily collectable. Sharing of case studies is encouraged. The

For peer review only

For peer review only - http://bmjopen.bmj.com/site/about/guidelines.xhtml

workshop directly informed NIHR's funding call. ambitious data-enabled trials at scale. There is the opportunity for the United Kingdom to build upon existing data science capabilities to identify, recruit and monitor patients in trials at scale.

For peer review only

For peer review only - http://bmjopen.bmj.com/site/about/guidelines.xhtml

INTRODUCTION

Clinical trials will continue to be the gold-standard for testing pharmacological, behavioural and policy interventions for the foreseeable future. The advent of the COVID-19 pandemic has raised the profile and importance of trials with the British public.[1] For a large majority of conditions, late-phase randomised controlled trials are commonly long-term endeavours which are increasingly expensive, particularly Industry-led trials. There is considerable overlap between the data that trialists need to assess impact of interventions on patients and what is already routinely collected in patient's health records. These routinely-routinely collected data are reflected in local, regional or national datasets, collectively labelled here as "electronic health records" (EHR) (**Box-1**). It is equally self-evident that safely making these routinely-routinely collected data available to researchers for purposeful access could decrease duplication of effort, reduce costs and may speed answers from some trials. The value of EHR in clinical trials is increasingly recognised by many organisations, including the United Kingdom (UK) Government as highlighted in the Life Sciences Industrial Strategy.[2,3]

The National Institute for Health Research (NIHR), Health Data Research UK (HDR UK) and the Clinical Practice Research Datalink (CPRD) (**Box-2**) brought together UK researchers for a one-day workshop in Oct-2018 to discuss how clinical trials might undergo a step-change in the UK through being "data-enabled". "Data-enabled trials" were defined for the workshop as introducing efficiencies in the following ways: improved study planning and feasibility assessments; more efficient recruitment, facilitated by accessing EHR to identify potential participants; efficient assessment of treatment benefits and potential harms by directly capturing routine EHR into the trial dataset; and more efficient study analyses and interpretation, using standard EHR variables. Many lessons from the workshop are also relevant to clinical trials more broadly.

For peer review only - http://bmjopen.bmj.com/site/about/guidelines.xhtml

Informed by this workshop, NIHR commissioned a cross-programme call for "Data-enabled trials"[4]. **Table-1** contains a list of data-enabled trials that were funded as a result of this call.

While there is recognition that challenges exist to accessing and using all sources of EHR, great strides have already been made realising the potential for using available EHR in innovative ways to support the delivery of patient consented studies. The workshop's spirit was therefore to focus on what had already been achievable, and to consider imaginable solutions to current challenges and further possibilities that could be developed in the future at scale. Speakers and facilitators were asked "to demonstrate existing capability and opportunities in the UK for undertaking data-enabled clinical trials, in particular showcasing the use of routine data to support different components of trial design and delivery, and regulatory considerations." The workshop, which was attended by researchers from around the UK, included plenary talks, panel discussions, group discussions and the presentation of selected case studies. The elements were broadly grouped as: Design and feasibility; Recruitment; Conduct and follow-up; Collecting key data on benefits and harms; and Analysis and interpretation. Here, we summarise and update some key issues that emerged in these areas in order to inform future discussions and research activities.

For peer review only - http://bmjopen.bmj.com/site/about/guidelines.xhtml
For peer review only

FINDINGS

Design and feasibility

Assessing the potential sufficiency of accrual is key to the planning and funding of all trials. Recruitment can be challenging both in prevalent and incident clinical conditions, particularly where potential participants are managing their chronic conditions in an environment away from active researchers. Robust and scalable processes to estimate the number of potential participants that might be suitable for a particular study need to be established.

Historically, complex care pathways made it difficult to estimate, and then locate, potential trial participants, because they might enter the systems in a number of ways. Use of evidence from the EHR, where these are available, enables a more realistic estimate of the achievable sample size within specific geographical regions, based on demographic and clinical information within EHRs. Key eligibility criteria such as demographic information, medication dose, laboratory results, timing of clinical events and comorbidities within the EHR allow rapid estimates of the number of potentially eligible people that can be flagged as suitable for a trial.

In the UK, general practitioners (GPs) are the gatekeepers to the NHS, and, as such, primary care records hold information that includes demographic and clinical data derived from consultations within primary care as well as secondary care visits and from other sources such as laboratory tests. Primary care records, covering a patient's medical history, are therefore a rich resource to carry out feasibility assessments and to locate patients for studies that might take place in a range of settings. Timeliness of data is important. The primary care data that CPRD receives, encompassing 25% of UK population, is updated daily, and it forms the basis for feasibility assessments CPRD routinely carries out for academic and industry led trials.[5] To further advance the use of EHR in supporting clinical trials, the National Health Service (NHS) DigiTrials

For peer review only - http://bmjopen.bmj.com/site/about/guidelines.xhtml

Health Data Research Hub for Clinical Trials was established by HDR UK in 2019 to facilitate feasibility assessments in England, initially using secondary care Hospital Episode Statistics (HES).[6,7]

The usual method of locating patients for many studies involves manual searches of patient records at each GP practice. The COPE study[8], a patient-consented, longitudinal cohort study, was one of the first studies in the UK exploring the use of EHR as a means of locating specific patients for enrolment into a study. The study, which sought to identify patients with chronic obstructive pulmonary disease (COPD) with prior exacerbation events (**Box-3**), demonstrated that it is possible to use a primary care EHR database to screen, locate and recruit participants for research. This method "provides access to a cohort of patients while minimizing input needed by GPs and allows researchers to examine healthcare usage and disease burden in more detail and in real-life settings".[8] CPRD carried out a centralised search for eligible patients in its database of pseudonymised patients, across a defined geographical region and GP's in CPRD's network were supplied with a list of potentially eligible patients based on the study inclusion and exclusion criteria. The GP then carried out a clinical review and invited potentially suitable patients to attend specific centres in which recruitment and collection of bespoke data could be completed. This intermediate clinical validation step involving the GP confirming a patient's eligibility, both facilitated locating high quality patients and maintained patient confidentiality.

A large proportion of trials fail to recruit to time and target,[9] and the funding wasted on these efforts may be better invested in modest pre-trial efforts to determine likely feasibility. Assessing potential numbers of patients can reasonably be done with near-real-time, longitudinal EHR (likely primary care datasets) or an administrative EHR record, like HES, which is cross-sectional and broad, but has a time-delay. Given the demonstrated value of using a EHR evidence-based approach to assessing feasibility, research funding bodies will need to give careful consideration to how such work-up activities can be supported as dedicated funding for research usually starts only after the work to assess feasibility has been attempted. Key points to emerge for study planning using EHR were the importance of using a validated algorithm to

For peer review only - http://bmjopen.bmj.com/site/about/guidelines.xhtml

search the data and the benefits of incorporating a clinical review to further refine the specificity of the search. These approaches can result in a much higher proportion of suitable and engaged patients presenting for detailed eligibility assessment at study sites.

Recruitment

Recruiting patients to research studies should be a standard part of routine practice. The majority of UK hospital trusts engage in research activity and this is increasingly true in primary care with 40% of GP practices in England participating in research in 2018/19.[10] However, for most practitioners, there is limited time to spend with patients, and often not enough time to explain research so lower burden approaches must be found. [11,12] Searches of GP patient records for patients potentially suitable for clinical studies isn't new. However, this is usually done by searching right through GP patient records on a local practice basis, which can be a burden for practice staff and introduce variability in interpretation of selection criteria. Centralised patient eligibility searches, such as those carried out by CPRD and North West eHealth, reduce the burden on practice staff by supplying the practice a list of potentially eligible patients located through standardised database searches against protocol inclusion and exclusion criteria.

Large-scale use of EHR for patient searches must take into account information governance regulations and the need to maintain patient confidentiality. One approach is to search pseudonymised patient data, where a patient's identity is unknown to the researchers, as is the case with CPRD recruitment services. Potentially suitable patients are re-identified by their GP, thereby maintaining confidentiality between the patient and the clinician. In some cases, specific regulatory approvals may be granted by to individual researchers to obtain access to identifiable EHR patient data in order directly invite them into a clinical trial. Alternative methods for recruiting patients into prospective trials include establishing a cohort of patients or volunteers who are willing to take part in trials, such as the NHS Scotland SHARE scheme[13] and COVID-19 vaccine trials portal,[14] – volunteer datasets may contain phenotype or genotype data not available in clinical or administrative datasets -- and pre-consenting trial participants to be re-approached

For peer review only - http://bmjopen.bmj.com/site/about/guidelines.xhtml

about other studies, such as those following on from the INTERVAL trial (NCT01610635) of blood donation timing.[15]

EHR are a tool that can be used to speed the pairing of willing patients with suitable trials.[16] DECIDE is a trail-blazing randomised pragmatic clinical effectiveness trial, involving a three-way partnership between academia, industry and CPRD, which recruited patients with Type-2 diabetes in a routine primary care setting, randomised to the licenced intervention or standard of care.[17] The study involved centralised searches of CPRD's primary care data to enable GPs in the CPRD network across all four UK nations to readily enrol suitable patients into the study when they presented for their routine diabetes checks. The novelty of this pragmatic clinical effectiveness study was that the study clinical data was captured directly from the patient's primary care EHR and flowed into CPRD's interventional research services platform (IRSP). The trial including randomisation was remotely managed via this central digital platform. The EHR derived study data was supplemented by electronic patient-reported outcomes collected by patients remotely logging into the IRSP. **Box-4** summarises the key lessons from DECIDE, using real-time EHR to facilitate recruitment and trial management. The DECIDE study demonstrates how it is possible to recruit patients across the UK into a randomised trial involving real-world Type-2 diabetic patients, based on changing laboratory HbA1c readings using dynamic data searches on primary care EHR data. This approach of using primary care data for time-bound patient recruitment, offers great potential and generalisability for Phase-II to Phase-IV patient recruitment, independent of relying on busy clinicians identifying patients presenting at clinic visits.

The majority of clinical trial protocols involve inclusion or exclusion variables that are time-dependent, for example timeframe from diagnoses, procedures or prescriptions. Furthermore, many trials will have a limited window of opportunity during which recruitment can take place for any individual potential participant. If patients are identified too late, the research invitation could arrive after it stopped being relevant; anecdotally, such irrelevant invitations have been confusing and upsetting to vulnerable

For peer review only - http://bmjopen.bmj.com/site/about/guidelines.xhtml

populations. In these circumstances, the EHR source used to located suitable patients for trial recruitment must contain patient information that is both current and dynamic.

Primary care data can provide a longitudinal and timely resource for locating patients suitable for both incident and prevalence trials. For example, CPRD's primary care records are updated daily and include hospital discharge information, so the system lends itself well to locating patients for trials taking place in primary or secondary care. GPs can also be alerted through system 'pop-ups' or dynamic daily refreshed patient lists, as for the DECIDE trial, which can support recruitment into incident trials for patients who present at the GP practice with acute or unmanaged conditions.

HES data can also be used to locate patients who have visited hospital and may be suitable for a trial. The ORION-4 trial is using a combination of HES data searches and patient questionnaires to recruit 15,000 patients from across the UK with atherosclerotic cardiovascular disease.[18] However, HES data has its limitation as it may only be available some months (often 3 months) after a patient hospital visit, it does not include a patient's full medical history and may not include all potential patients, such where the condition was not severe enough to require a hospitalisation eg mild stroke. Some hospitals have developed their own EHR systems that support recruitment of patients presenting with acute conditions. However, there are currently no cross-hospital, inter-operable electronic systems in use in secondary care that support dynamic recruitment.

Conduct and follow-up

Access to routine EHR for individuals participating in a trial to monitor their important and relevant health outcomes has been a fundamental challenge for many trials that have attempted access late in the trial. This has often involved a lengthy application process via a changing approval system, frequently resulting in a considerable lag between requesting and accessing data. Many researchers have persisted,

For peer review only - http://bmjopen.bmj.com/site/about/guidelines.xhtml

recognising the huge potential for efficient use of EHR in the conduct and follow-up of trials, particularly large-scale national trials.

ASCEND[19,20] was a UK-wide trial in 15,500 people with diabetes, evaluating the effect of aspirin and omega-3 fatty acids on the risk of cardiovascular events. The trial, whose key design features are summarised in **Box-5**, used mail-based methods for recruitment, intervention delivery and follow-up, thereby avoiding the need for dedicated study sites. Initially, follow-up was by a paper-based questionnaire, but, in the latter years of the trial, an online form was available for follow-up for those who preferred. Towards the end of the 7.5 year follow-up period, access to HES data was granted, which allowed missing outcome data to be recorded for participants who had not returned questionnaires. Preliminary unpublished comparisons in ASCEND between the questionnaire data and the EHR data showed good concordance, with analyses of the primary trial outcomes producing similar results irrespective of the data source used. [Personal communication]

Although the trial was beneficial for patients avoiding the need to travel to trial sites, it was labour-intensive for the study team, involving mailing and phoning thousands of participants and their GPs. Considerable efficiencies could have been possible if the HES data had been used as the main source of outcome information from the outset. Further benefits could be derived by accessing primary care data, as some cardiovascular events do not require hospitalisation and are consequently not captured in the HES data. The HES dataset is usually at least 3 months old. However, the 'rawer' form of HES, called Secondary Uses Service (SUS+) is being used in the RECOVERY trial to enable faster, albeit *in extremis,* assessment of health outcomes for patients with severe COVID-19 infection.

EHR can be used to assess the short-and long-term impact of an intervention on health outcomes of individual patients at participating sites. This can be through a patient-level intervention or via a randomised cluster trial where the intervention is applied at a site level. One such example is the REDUCE

For peer review only - http://bmjopen.bmj.com/site/about/guidelines.xhtml

cluster randomised trial (ISRCTN95232781) embedded with the CPRD GP practice research network which used a digital intervention versus standard of care to attempt to reduce unnecessary use of antibiotics for respiratory infections in primary care. In addition to using the EHR to monitor prescribing rates, EHR data was used to monitor patient health outcomes in the intervention and standard of care GP practice arms of the trial.[11] Findings from ASCEND[19,20], COPE[8] and DECIDE[11] support the advantages gained for data collection and follow-up by using EHR from the outset for patients recruitment.

The Office for National Statistics system has flagged people as participating in each of around 200 approved trials so that mortality data could be provided to the trial in due course. All EHR systems need to include this ability to flag for participation in a specific trial. Without flagging, trialists and EHR providers need to go through the linkage process. This increases costs against limited budgets, is disproportionately effortful and exposes a repeated risk of linkage errors. Both Public Health England (PHE) National Cancer Registration and Analysis Service (NCRAS) and NHS Digital are piloting approaches to trial flagging.

Collecting outcome data on benefits and harms

Access to timely data is key for any trial, and trialists must be able to report on safety and key outcome measures in a suitable timescale, including for the final analyses and for Data Monitoring Committee (DMC) review of interim analyses. Trials must meet the stringent regulatory timelines set out in UK law, which includes reporting to the competent authority, appropriate suspected unexpected serious adverse reactions (SUSAR), within short timelines that starts when the sponsor of the trial receives the information from the clinical site.

The timely capture and reporting of certain safety outcome measures are critically important for the Clinical Trials of Investigational Medicinal Products (CTIMP) trials run under the clinical trials regulations, where a subset of Serious Adverse Events (SAE) must be reported to the regulator within a short, prescribed timescale of 7 or 15 days. Researchers must quickly determine whether the SAE is a “reaction”

For peer review only - http://bmjopen.bmj.com/site/about/guidelines.xhtml

(SAR) and, if so, whether this reaction is an "unexpected" (SUSAR) according to clearly-defined Reference Safety Information. SAR and SUSAR are more likely in some treatment settings e.g. cancer chemotherapy trials. "Seriousness" definitions include, among others, "hospitalisation" or "prolongation of hospitalisation" which may or may not be captured across different EHR systems.

Frequency of updates to routine EHR data depends on where in the system the data are collected. Primary care records, such as those received by CPRD, are updated daily, but administrative-focused EHR datasets, such as HES, may provide only a snapshot of data from some months ago. Trials using only periodically-updated, routinely-collected EHR generally cannot rely on these data for reporting purposes in regulatory timescales, in particular for safety monitoring. It is a key responsibility of researchers to understand the strengths and limitations of any EHR data source, including its suitability to meet reporting requirements. A future involving interconnected real-time systems would allow investigators to reliably detect and report appropriate events.

The Salford Lung Studies were two industry-sponsored, late-phase randomised controlled trials (RCTs) which were the first studies in the world to have evaluated the effectiveness of a pre-licenced medicine in a real-world setting. The study capitalised on access to primary care EHR, as well as community pharmacy data and electronic secondary care patient records embedded the regional hospitals. Altogether 7200 patients with COPD or Asthma were monitoring in near real-time for safety and outcomes using city wide linked EHR.[21 22] The study team met with the regulators to agree the outcome measure data and safety alerting and reporting systems, which were predicated entirely on capture of routine data from near real-time primary and secondary care systems. As outlined in **Box-6**, the studies successfully demonstrated that it is possible to run complex large-scale trials with configurable near real-time safety monitoring systems that meet regulatory standards

For peer review only - http://bmjopen.bmj.com/site/about/guidelines.xhtml

It is important to check up-front in efficacy and effectiveness trials that may be used for regulatory submission whether routinely-collected EHR would be accepted as source data by the relevant regulatory authorities. The Medicines and Healthcare products Regulatory Agency (MHRA) now considers using source data directly from each hospital's EHR standard following digitalisation of health records. GCP inspections performed by the MHRA in the UK are performed against the requirements of the UK Clinical Trials Regulations, irrespective of whether the source data is electronic or paper in format. The MHRA recognises that there is no standardised method of recording clinical source data, especially in international multi-centre trials.[Personal communication] Variations across sites include how many electronic systems make up the EHR, and how clinical data are reported through local laboratory electronic systems. It is therefore recommended each sponsor reviews the type of data required for their trial and how it would be recorded at site, including the various electronic systems that capture the source data and whether they are fit for purpose.

The source data and its underpinning EHR system needs to follow the "ALCOA+ principles" to be attributable, legible, contemporaneous, original and accurate, as well as complete, consistent, enduring and available.[23] This ensures the data are reliable and that the electronic system is fit for its intended purpose. This should include appropriate access levels for investigator site trial teams, sponsor representatives and Government Regulators, and is supported by the International Conference on Harmonisation (ICH) E6 R2 Addendum. Careful work is required to ensure that systems which collate data from multiple healthcare systems (eg HES or registry data) follow these principles. Review of the data on regulatory inspection by the MHRA includes assessing whether the trial data can be reconstructed, and that the above requirements are met. Reconstruction includes assessing whether the system has an associated audit trail verifying who completed assessments and when, and the ability to reconstruct any changes to the source data. Source data verification performed during MHRA inspections is always done on a risk-based approach, with a focus on the critical data for the trial and compliance with the clinical

For peer review only - http://bmjopen.bmj.com/site/about/guidelines.xhtml

trials regulations, recognising that EHR systems are often designed with standard care not clinical trials in mind. The same risk-based approach should be taken by sponsors auditing their own trial data.

Analysis and interpretation

Many researchers desire collecting as much data as possible for their trial through routine EHR sources, thereby minimising the collection of data through trial-specific means. Reviewing metadata or de-identified samples of data, broadly representing the data that should be available for the sort of patients that could join a future trial, is recommended to determine which datasets include suitable variables to monitor health outcomes from EHR sources. In the case of cancer, PHE has developed Simulacrum to imitate some of the data held by NCRAS.[24] The Health Data Research Innovation Gateway has been initiated by HDR UK as an open repository of data with associated metadata, to accelerate discovery and access to existing health data research resources.[25] Some argue that a common repository of data quality checks may be useful for researchers running data-enabled trials. Carrying out data utility assessments are a practical method of determining where EHR could substitute for trial-specific collection in future trials.[26,27]

Any single EHR dataset is not likely to collect all the necessary information, in a timely and consistent manner, that can be used to evaluate health outcomes across all trial arms. **Box-7** sets out some of the lessons learned during the SANAD-II trial.[28,29] This trial for patients with epilepsy found that seizures, key to any epilepsy core outcome set, had not been well recorded in the accessible EHR sources. Initiatives such as the COMET (Core Outcome Measures in Effectiveness Trials) are seeking to develop sets of core outcome measures (COS) that are suitable to support RCTs, as well as audit, research and routine care. Researchers developing COS should strive to make them as similar as possible to both the information required in routine practice and the key outcomes needed by regulatory reviewers.

For peer review only - http://bmjopen.bmj.com/site/about/guidelines.xhtml

Routinely-collected datasets have contrasting and complementary strengths: hospital EHR datasets are rich in specific areas, but lack the wider medical history for each patients; GP EHR datasets capture the broader, longitudinal medical history, but not all the specialist depth; and registry datasets may have rich healthcare data, but for only a narrow patient sub-group; and central administrative datasets collating information across healthcare systems may be wide, but not deep. Therefore, it may be necessary to use multiple datasets to analyse trial outcomes and these datasets may need to be linked. Well established processes exist to link data to trials, although delays can emerge from governance and data access issues.

Data linkage can be used to validate data collected on an individual trial participant from trial-specific source or other routine datasets. The comparisons of multiple sources can support assessment of generalisability and external validity for future research. The use of alternative data sources may address any concerns about the representativeness of patients in a trial in comparison to those who, for whatever reason, did not participate (e.g. trial not open in the area), including checking that the participants are broadly representative of the target population. An example of this is where primary data were used to validate the outcomes of the real-world Salford Lung Studies, in the broader UK population thereby ruling out any bias that may be introduced due to a regional Hawthorn effect.[30] For treatments that are widely available outside of the research setting, researchers may assess external validity or representativeness of the patient populations if data can be requested for an equivalent non-randomised anonymised cohort of patients.[11]

For trials that combine multiple sources of data, trialists must pre-specify what will be done in the instance of discrepancy between sources: which source would take primacy, who makes that decision, how checking will be done, and whether there is any opportunity to correct one of the data sources, if required, in a manner consistent with data protection regulations about holding accurate data.

For peer review only - http://bmjopen.bmj.com/site/about/guidelines.xhtml

It is critically important to ensure that the correct EHR variables are both well understood and used in the right way to calculate the relevant outcome measures. Using multiple sources with differences in terminology and structure requires careful, prospective analytic approaches to derive meaningful information about the safety and efficacy of treatments.

For peer review only

For peer review only - http://bmjopen.bmj.com/site/about/guidelines.xhtml

DISCUSSION

NHS datasets provide a record of population-level health data and afford rich opportunities for improving health through evidence-based medicine. There is a recognition that the UK can grasp these opportunities and address the challenges required to usher in a new generation of more efficient and cost-effective clinical trials. Many of these trials will be "data-enabled trials", with routinely-collected EHR driving their design and feasibility, their screening and recruitment and their management and analysis. Others will use EHR for more established practices such as long-term participant follow-up. The workshop demonstrated that EHR can readily be used to assess trial feasibility and to facilitate patient recruitment from Phase-II to Phase-IV. However, attendees sensed that the short-term emphasis for conducting "fully data-enabled trials" should be on licenced treatments in different populations or for new indications, where outcome measures are readily collectable from the EHR. Trials like the Salford Lung Studies and DECIDE serve as vanguards demonstrating the application of real-world evidence in fully data-enabled clinical trials.[17,21,22]

EHR data are collected in different formats and frequency depending on their purpose and provenance. There are multiple EHR–collating-and-holding bodies within the UK, but no one body can provide access to all sources of EHR data. This situation results in a complicated web of access routes to datasets, differing in scope, method of collection, currency of data, quality controls, availability of metadata and connection to additional linked information. It is currently estimated that fewer than 5% of trials in the UK successfully access routinely-collected datasets to support trial execution,[31] much lower than the proportion of trials planning to access such data at the grant application stage.[32] Therefore, more is clearly needed to increase confidence and capabilities in the use of routine health data across the UK clinical trials community. In the short-term, route maps depicting these dataset details and access methods would be welcomed. In the long-term, the trials community would be keen to see intra-operable EHR systems providing simple access to up-to-date datasets to facilitate trials: intra-operability and reusability emerged repeatedly as themes throughout the workshop. There are complex and varying coding systems across the

For peer review only - http://bmjopen.bmj.com/site/about/guidelines.xhtml

various healthcare practice settings for which clarity is required. The UK Secretary of State has been aware of the need for better data standards in healthcare and has started to put NHS quality data into dashboards.[33] Many data items are held in free-text so sophisticated natural language processing (NLP) approaches will be required to extract coherent, targeted information, whilst upholding confidentiality standards. HDR UK's Health Data Research Innovation Gateway have has progressed towards making more accessible the datasets routinely available datasets more accessible for a range of research uses.[34] although initially this resource is more likely to facilitate re-use of data from trials rather than supporting recruitment and conduct of new trials.

Gaining the necessary permissions to access EHR data causes challenges for many researchers, particularly when the interpretation of patient consent has changed over time with the strengthening information governance requirements. Consent from trial participants to access EHR data is taken in many trials only with the intention of accessing long-term outcome measures, so researchers may not submit the relevant applications until the linked data are needed, late in the trial. Unfortunately, expectations around consent might have changed by this time point, complicating the process. Researchers are therefore strongly recommended to access EHR data from early in the trial and for trial consent forms to prospectively cover broad and enduring consent for access to routine EHR as a matter of course in order to facilitate research.[35]By embedding the EHR from the design stage, with appropriate consent, data-enabled trials avoid this issue. A more streamlined approach to governance with greater consistency and clarity in terminology and requirements for enduring patient consent for research may facilitate future data access for all trials. Where additional data are required and collected for a clinical trial, there may be potential to augment the patient's EHR with this information, such as results of genetic or genomic tests or patient-reported outcome measures.

Clinical trials are expensive and slow to plan and complete, and it is necessary to reduce research waste through all possible methods.[36-39] Trial design takes at least 6 months, yet is often insufficiently resourced,

For peer review only - http://bmjopen.bmj.com/site/about/guidelines.xhtml

relying on "own account" work taken at the risk of the sponsor (usually a higher education institute) prior to grant award. A substantial proportion of trials are delayed or do not reach their recruitment target;[9,40] and a considerable number require at least one protocol amendment.[41] EHR offer opportunities to better plan clinical trials by characterising the populations and their treatment or diagnostic pathways as well as assessing the trial feasibility based around specific geographies.[8] The proportion of patients approached and recruited to trials also varies according to different demographics such as ethnicity or age.[42,43] Using EHR data to identify specific patient pools will enable trials in under-served populations or where disease burden is the greatest, thereby democratising research and increasing the potential recruitment efficiencies.[44] Increasing the representativeness of trial participants also enhances external validity of the findings, especially as guidance such as the INCLUDE ("Innovations in Clinical Trial Design and Delivery for the Under-served") framework is now available on the issues to consider to be more inclusive on attributes such as ethnicity.[45] UK-wide representative population coverage, through CPRD, has demonstrated the proof of principle of successfully recruiting a demographically representative patient population into the pragmatic DECIDE in Type-2 diabetes trial, through centralised primary care patient data eligibility searches [Personal communication]. Virtually all of the UK population is registered with a GP practice and GPs are gatekeepers to the NHS, often managing chronic conditions that have been diagnosed in secondary care. Barriers to GPs' participation in primary health care research are well recognised.[46,47] We anticipate that new, data-enabled methods will increase awareness of research participation for both patients and GPs. The role of GPs acting as potential "gatekeepers" to research will be an important issue, recognising that ethics committees or regulators may require the approval of a clinician who has responsibility for the patient in some studies. With their rich, longitudinal medical history, primary care datasets represent an excellent source of data to locate suitable patients for trials based in both primary and secondary care settings. A strong desire is a single resource which brings together all primary care data within and across all four UK nations.

For peer review only - http://bmjopen.bmj.com/site/about/guidelines.xhtml

Validation of the choice of EHR codes used in patient cohort searches enables greater specificity of patient searches, leading to higher patient conversion rates into and less time lost by patients and trial teams at the trial screening stage.[Personal communication] Clinical validation of potentially suitable patients initially flagged by EHR searches, further refines the patient search, meaning that the patients approached to take part are more likely to pass eligibility screening, which improves the efficiency of patient recruitment. Assessment of the variables contained within the EHR at the trial design stage enables a better understanding of which variables can be obtained from routinely-collected data, removing duplication of collection. Moving from only trial-specific data collection to more widespread use of EHR within trials, should improve the cost-effectiveness of trial funding. Double-blinding and placebo-controls are more difficult aspects of conduct to operationalise in routine EHR settings, but may be offset by robust data, careful protocols and objective outcome measures. Each trial's risk assessment should be clear on any risks associated with the use of routine EHR data. Formal regulatory advice meetings may provide clarification of the proposed approaches, including monitoring and data verification. SAEs require construction by the investigators of an accompanying narrative which may require detail beyond that collected by the research team access routinely-collected EHR source, but which should be available to the responsible investigator.

Methodological engagement, such as from MRC-NIHR Trials Methodology Research Partnership[48] will help to assess the settings for which using routinely-collected data are, or are not, more suitable than trial-specific data collection. A Study Within A Trial (SWAT) approach[49-51] can efficiently assess particular elements of EHR trials, such as from the WOSCOPS trial.[52,53] Collation of findings from retrospective comparisons will elucidate issues for prospective clarification and highlight challenges to address around better data collection.[26]

The impact of 2020's SARS-COV-2 pandemic was keenly felt by clinical trials with many hindered by a necessary pause, postponement or termination of recruitment and/or treatment. Routine hospital

For peer review only - http://bmjopen.bmj.com/site/about/guidelines.xhtml

appointments became virtual and many research assessments had to follow. While trials teams could pivot to collect data online directly from participants, postponed or cancelled scans and tests will cause analysis and interpretation issues through the trial literature for many years which will need to be reported systematically using the extension to CONSORT (Consolidated Standard for Reporting Trials) standards currently in development. National strategic prioritisation of trials opened doors to routinely-collected data with uncommon rapidity. This may allow the development of new access routes to data for trials and more wide-spread adoption by the research community of EHR-based methodology within trial delivery.

The UK has led by example in patients and public involvement and engagement (PPIE) for trials,[54,55] and key funding bodies support clear PPIE. Patient ambassadors may be particularly helpful in informing the development of data-enabled trials. Locating participants via their EHR will enable participants to be informed of the trial's results, which, surprisingly, still does not always happen, although there is limited information available on best practice to inform participants; the Show Respect study is addressing this.[56] The StatinWISE study (NCT02781064), a non-randomised cohort N-of-1 study of stopping statins in patients who have had heart attacks presented each patient a booklet of their own results before the study reported overall findings.[57] There is a clear need to engage the public on clinical trials, sensitising communities to research, and with a specific focus on the capacity of EHR to provide better outcomes.

This workshop showcased key successes and high-profile case studies (**Boxes 3-7**) that have answered questions where EHR data could map well to the trial questions, supplemented by some trial-specific collection. Over-interpretation of the clinical trials regulations can be risk-averse and suffocate innovation. Simplicity in implementation need not be interpreted as basic in ambition: straightforward protocols can still address a number of key clinical questions simultaneously.[1,58] Knowledge sharing and capability-building exercises now should facilitate future data-enabled trials. Trialists are getting better at writing up their findings and, increasingly, their designs, but further effort is required in writing up their practical

For peer review only - http://bmjopen.bmj.com/site/about/guidelines.xhtml

implementation experiences. Best practice and case studies, reflecting on successes and challenges, should be shared, including in the threaded publications from NIHR HTA (Health Technology Assessment) monographs[59] or a new, dedicated national forum.

Five years previously, NIHR launched a call for trials using routinely collected data which elicited a modest response form research community. Stimulating research using routinely collected clinical data has remained a sustained interest of NIHR and launching out from this workshop, NIHR announced a new call for data-enabled trials.[4] NIHR committees reviewed 18 applications and the funded trials were confirmed in 2020 (**Table-1**).

Routinely-collected EHR offer great potential for faster, more efficient and less expensive trials reaching a broader catchment of the UK population. Wider adoption of EHR-based approaches and experiences from these studies, such as those funded from the NIHR data-enabled trial call, will provide the evidence to assess where EHR enablement brings benefits to trial execution and follow-up. Key recommendations from the workshop are presented in **Table_2**, many of which are now being addressed. Using EHR data for trials can be done and could be done at a greater scale than is the current practice. The desire to access and efficiently use these data is widely discussed and this workshop demonstrated that this aspiration is possible. The opportunities to learn and harness the power of information and technology to improve healthcare outcomes for everyone has never been better.

For peer review only - http://bmjopen.bmj.com/site/about/guidelines.xhtml

BOXES, TABLES AND FIGURES

Box-1: What are routinely-collected electronic health records?

There are vast amounts of data routinely being collected on patients, for example, in patient electronic health records (EHR), as well as disease and patient registries. Such data is commonly called real-world data (RWD), reflecting that it is routinely collected while patients go about their regular lives, as opposed to being specifically collected in a clinical trial. Because the NHS is a universal healthcare provider, free at point of care, the NHS EHR offers the possibility to collect the longitudinal medical history of 98% of the UK population who uses the NHS. EHR therefore provide an excellent research tool for studying population health. Primary care EHR are recorded by GPs and contain longitudinal demographic and clinical patient information encompassing symptoms, diagnoses, prescribing, laboratory tests and data from hospital visits and admissions. Primary care EHR can be collected and made available for research in near real-time. Secondary care records capture interactions that take place in a specific hospital and are event based, not longitudinal. Secondary care records are coded for reimbursement purposes, and not usually by clinicians. There is a time lag between patient hospital visits and secondary care EHR data being made available for research and NHS planning purposes.

For peer review only

For peer review only - http://bmjopen.bmj.com/site/about/guidelines.xhtml

Box-2: NIHR, HDR UK and CPRD

**National Institute for Health Research**<br>NIHR, a virtual organisation, is the research arm of the NHS. It comprises an Academy of trainees and senior investigators, and systems to make research processing easier and faster, including infrastructure, such as the clinical research network that plays a vital role in delivering research, and a number of research funding programmes. These include, for example, the NIHR Health Technology Assessment Programme, NIHR Health Services and Delivery Research and the NIHR Public Health Programme that fund prioritised health research for the public on behalf of the Department of Health and Social Care.
**Health Data Research UK**<br>HDR UK is the national institute for health data science. The institute, was established in 2018 as a joint strategic investment[1] with an aim to "unite the UK's health data to make discoveries that improve people's lives". Through initially six collaborative research sites across the UK and expanded to eight sites and 7 specialist HDR Hubs, the Health Data Research Innovation Gateway and Health Data Research Alliance, the Institute develops advanced health data science tools and technologies to analyse large-scale and diverse health data to solve complex health challenges. One priority themes is "Better, faster, and more efficient clinical trials".
**Clinical Practice Research Datalink**<br>CPRD is jointly sponsored by the Medicines and Healthcare products Regulatory Agency (MHRA) and NIHR, and is part of the Department of Health and Social Care. CPRD is the UK Government's dedicated real-world research service, which collects de-identified patient data on a daily basis from a network of one in every five GP practices across the UK, encompassing 25% of the UK population. The CPRD database includes longitudinal primary care records on a total 60 million patients collected over CPRD's 30 year history, with a median patient follow-up time of 10 years. Approximately 40% of CPRD's GP practice network are research-active.[16]

[1] Health Data Research UK is funded by the Medical Research Council, the health research departments of England, Scotland, Wales and Northern Ireland (National Institute for Health Research (NIHR), Chief Scientist Office (CSO), Health and Care Research Wales, HSC Research and Development respectively), the Economic and Social Research Council (ESRC), the Engineering and Physical Sciences Research Council (EPSRC), the British Heart Foundation and Wellcome.

For peer review only - http://bmjopen.bmj.com/site/about/guidelines.xhtml

For peer review only

For peer review only - http://bmjopen.bmj.com/site/about/guidelines.xhtml

Box-3: Key points from COPE study

**Study name (registration)**	COPE (Characterisation of COPD Exacerbations using Environmental Exposure Modelling)[8]
**Sponsorship**	Academic – Imperial, MRC funded
**Clinical setting**	Chronic obstructive pulmonary in primary care, UK only: national
**Design**	Patient-consented longitudinal cohort study (non-randomised)
**Size**	N=130
**Key outcome measure**	COPD exacerbations
**Rationale for presentation**	Design and feasibility
**Key discussion points**	The aim of the COPE study was to develop a method for predicting COPD exacerbations as a result of environmental exposures. Quality algorithmic query specification to identify patient cohorts in EHR relies on data quality. The algorithm for COPE was validated in a previous study by comparing the EHR records with additional information provided by GPs.[60] The validated query was applied centrally to search for patients in the CPRD database. GPs were then contacted and asked to take part in confirming patient eligibility from a pre-screened patient list.<br>Centralised searching of the EHR greatly minimised GP screening time in confirming patient eligibility. It was also possible for a GP to exclude patients who may not be suitable to take part (e.g. due to caring for a sick relative). No algorithm could reliably account for these types of patient exclusions.<br>Lessons from the study were that it is feasible to recruit patients from EHR and readily collect data for long-term follow-up, although this method does depend on GP participation. Using EHR meant far less effort than manual screening of GP records. Because of the specificity of the EHR and clinical validation steps, more appropriate patients were approached which reduced time needed for patient screening. Patients approached were keen to be involved in the study and as a result the enrolment was higher than using conventional methods.

For peer review only - http://bmjopen.bmj.com/site/about/guidelines.xhtml

Box-4: Key points from DECIDE trial

**Trial name (registration)**	DECIDE: Pragmatic Randomised 104 Week Multicentre Trial to Evaluate the Comparative Effectiveness of dapagliflozin and Standard of Care in Type-2 Diabetes[17] (NCT02616666)
**Sponsorship**	Industry then academic -- AstraZeneca then Liverpool
**Clinical setting**	Type-2 diabetes mellitus on metformin and needing better glucose control in primary care setting, UK only: national
**Design**	Phase 4, randomised, open label, comparator trial
**Size**	N>800 (ongoing)
**Key outcome measure**	Clinical success (glucose control) at 12 months
**Rationale for presentation**	Recruitment
**Key discussion points**	DECIDE is a clinical effectiveness trial comparing dapagliflozin, a SGLT2 (Sodium-glucose co-transporter-2) inhibitor, to standard care (alternative medications such as sulfonylureas and DPP4 (Dipeptidyl-peptidase 4) inhibitors) in people with Type-2 diabetes requiring second-line pharmacological treatment (after metformin). There was a limited window in which patients need to be approached before treatment is changed.<br>CPRD managed the trial using their interventional research services platform which supported GPs and patients logging into the system. Algorithms were developed to carry out daily searches of primary care EHR for potentially eligible patients meeting pre-specified criteria. The primary care team then reviewed the search list and enrol eligible patients into the study at their next routine scheduled visit.<br>Most of the data required for the trial was available in CPRD's routinely-collected dataset, thereby facilitating collection of primary and secondary outcome measures as well as identification and recruitment. Patient-reported outcome measures were collected electronically. Trial monitoring could therefore be carried out centrally.<br>Lessons from the trial are that EHR search algorithms are easy to set up but feasibility assessments should take account of variability of coding and real-world clinical practice. Recruiting patients via EHR is achievable and allows easy collection of long-term data for follow-up but as with all trials it depends on GP and patient participation and engagement. Data-enablement and platform technology allows real-time adaptation to real-time conditions.

For peer review only - http://bmjopen.bmj.com/site/about/guidelines.xhtml

Box-5: Key points from ASCEND trial

**Trial name (registration)**	ASCEND: A Study of Cardiovascular Events iN Diabetes [19,20] (NCT00135226)
**Sponsorship**	Academic – Oxford
**Clinical setting**	Diabetes mellitus, UK only: national, mail-based methods – no study sites
**Design**	2 x 2 factorial design randomised placebo-controlled trial (Phase 4)
**Size**	N=15000
**Key outcome measure**	Cardiovascular events
**Rationale for presentation**	Recruitment, conduct and follow-up
**Key discussion points**	ASCEND used highly streamlined mail-based methods to identify, recruit and follow-up (for an average of 7.5 years) 15,500 UK patients, making it one of the longest-duration and largest ever trials in diabetes.<br>Participants completed 6-monthly follow-up questionnaires, initially using paper and paper but later moving to an online system for those who wished.<br>The trialists gained access to HES data during the closing stages of the study, allowing additional follow-up information to be gained for those who had not returned a recent questionnaire. Subsequent analyses of the HES data compared with questionnaire data showed good concordance, suggesting that, in the UK, HES data alone could be used as a highly efficient means of follow-up for cardiovascular trials in the future.

For peer review only - http://bmjopen.bmj.com/site/about/guidelines.xhtml

Box-6: Key points from Salford Lung Study

**Trial name (registration)**	Salford Lung Study[21] (Extension = NCT03152669)
**Sponsorship**	Industry – GlaxoSmithKline
**Clinical setting**	Chronic obstructive pulmonary, UK only: regional
**Design**	Early-phase, cluster randomised trial run in one geographic location
**Size**	N=7200
**Key outcome measure**	COPD exacerbation rates
**Rationale for presentation**	Conduct and follow-up, collecting data on benefits and harms
**Key discussion points**	The study was the first in the world to have evaluated the effectiveness and safety of a pre-licenced medicine compared with standard of care when used in every day clinical practice in patients with Chronic obstructive pulmonary disease (COPD). Patients were randomised at their routine GP respiratory review visit and constant monitored through the 12 month study via real time data collection from GP and hospitals. Data was also collected from participating community pharmacies and from Office For National Statistics (ONS) mortality and Secondary User Service national datasets and an out of hours phone service. A safety alerting and reporting system was established based on serious adverse events being initially flagged in the EHR followed by review by the safety team and the principal investigator prior to submission to the sponsor as appropriate.<br>Lessons learnt were to ensure at the design phase that all the safety and endpoints of the study could be readily captured and the importance of early engagement with the regulators and National Institute of Health and Care Excellence (NICE). The study demonstrated that safety monitoring in this type of real wold study was effective, could be done close to real time and was highly configurable. Widespread adoption could improve safety and such systems lend themselves to novel trial designs.

For peer review only

For peer review only - http://bmjopen.bmj.com/site/about/guidelines.xhtml

Box-7: Key points from SANAD-II trial

**Trial name (registration)**	SANAD-II[28,29] (ISRCTN30294119)
**Sponsorship**	Academic – Liverpool
**Clinical setting**	Epilepsy
**Design**	Randomised controlled trial
**Size**	N=1510
**Key outcome measure**	Time to 12 months without seizure
**Rationale for presentation**	Analysis and interpretation
**Key discussion points**	The SANAD-II (Standard and New Antiepileptic Drugs) trial. SANAD-II is a pragmatic, UK, multicentre, Phase IV randomised controlled trial (RCT) funded by the National Institute for Health Research (NIHR) Health Technology Assessment (HTA) programme, assessing the clinical and cost-effectiveness of a number of antiepileptic drugs as first-line treatments for newly diagnosed epilepsy. The primary outcome measure related to a period without seizures. These data were recorded on Case Report Forms (CRFs) by the treating clinical team at outpatient visits.<br>In a study of 98 patients, the team found the seizures were poorly recorded in EHR. Using only EHR data estimated that 74% of patients had spent one year seizure-free, whereas using only CRF data estimated that only 46% of patients had spent one year seizure-free. This is a clinically important difference. Researchers need to be able to trust in the completeness of EHR data to use them in reliable analyses or to understand how these data might supplement CRFs. This example cautions against naïve use of routinely-collected EHR data.

For peer review only

For peer review only - http://bmjopen.bmj.com/site/about/guidelines.xhtml

Table-1: Trials funded in Summer-2020 by first round of NIHR Data-Enabled Trials Call

NIHR panel	NIHR Ref	Trial title	Chief Investigator (Co-Chief Investigator)	Trials Unit	Planned start
HTA	NIHR130508	**UK-ROX:** Evaluating the clinical and cost-effectiveness of a conservative approach to oxygen therapy for invasively ventilated adults in intensive care	Daniel Martin	The Intensive Care National Audit and Research Centre	Q3-2020
HTA	NIHR130280	**DaRe2THINK:** Preventing stroke, premature death and cognitive decline in a broader community of patients with atrial fibrillation using healthcare data for pragmatic research	Dipak Kotecha	Birmingham Clinical Trials Unit	Q4-2020
HTA	NIHR130573	**VITALL** Volatile vs Total intravenous Anaesthesia for major non-cardiac surgery – a pragmatic randomised controlled trial	Joyce Yeung	Warwick Clinical Trials Unit	Q4-2020
HTA	NIHR130548	**ASYMPTOMATIC:** Assessing Symptom-driven versus Maintenance Preventer Therapy for the Outpatient Management of Asthma In Children	Ian Sinha (Paula Willliamson)	Liverpool Clinical Trials Centre	Q1-2021
HS&DR	NIHR130075	In-silico trials of targeted screening for abdominal aortic aneurysm using linked healthcare data: can the efficiency of the NHS Abdominal Aortic Aneurysm Screening Programme be improved whilst maintaining publicly acceptable levels of disease detection in an ethically acceptable manner	Matthew Bown	n/a	Q4-2021
HS&DR	NIHR130581	Cluster randomised trial to improve antibiotic prescribing in primary care: individualised knowledge support during consultation for general practitioners and patients	Tjeerd van Staa	Manchester Clinical Trials Unit	Q3-2020
HS&DR	NIHR130107	**ATHENA-M:** Observational study of age, test threshold and frequency on English national mammography screening outcomes	Sian Taylor-Phillips	n/a	Q4-2020

Key: HTA = Health Technology Assessment, HS&DR = Health Services and Delivery Research, PHR = Public Health Research
Note: 18 applications were submitted to the NIHR data enabled trials call across 3 research committees. 4 of 9 applications to HTA accepted for funding; 2 of 5 applications to HS&DR accepted for funding with 1 additional application still to be decided; 0 of 3 application to Public Health Research accepted for funding

For peer review only - http://bmjopen.bmj.com/site/about/guidelines.xhtml

Table-2: Recommendations for data-enabled trials

- Wider adoption of data-enabled methods for clinical trials
- Build in methodology questions into the trial protocol eg conversion rates for identification to recruitment; refining the process for identifying potential patients; understanding event rates for the target population
- Early engagement of regulators with regards to monitoring and data verification
- Patients and public involvement to robustly inform data-enabled trials
- Increased inter-operability of electronic health record (EHR) systems
- Clear map for access to EHR: what is captured where, what do the data mean and how can they be used
- Consistency between core outcome sets used for research and routine practice
- Wider sharing and reporting of EHR code-lists to support recruitment and outcome measures
- Sharing of detailed examples of success stories
- Capacity- and capability-building for future data-enabled trials

For peer review only

For peer review only - http://bmjopen.bmj.com/site/about/guidelines.xhtml

ACRONYM LIST

Acronym	Name
ALCOA+	Attributable, Legible, Contemporaneous, Original And Accurate, And Consistent, Enduring And Available
COMET	Core Outcome Measures In Effectiveness Trials
CONSORT	Consolidated Standard For Reporting Trials
COPD	Chronic obstructive pulmonary disease
COS	Core Outcome Set
CPRD	Clinical Practice Research Datalink
CRFs	Case Report Forms
CTIMP	Clinical Trials Of Investigational Medicinal Products
DPP4	Dipeptidyl-peptidase 4
DMC	Data Monitoring Committee
EHR	Electronic Healthcare Records
GP	General Practitioners
HDR UK	Health Data Research UK
HES	Hospital Episode Statistics
HTA	Health Technology Assessment
ICH	International Conference On Harmonisation
INCLUDE	Innovations In Clinical Trial Design And Delivery For The Under-Served
ISRP	Interventional Research Services Platform
MHRA	Medicines And Healthcare products Regulatory Agency
NCRAS	National Cancer Registration And Analysis Service
NHS	National Health Service
NICE	National Institute of Health and Care Excellence
NIHR	National Institute Of Health Research
ONS	Office For National Statistics
PHE	Public Health England
PPIE	Public Involvement And Engagement
RCT	Randomised Controlled Trial
RWD	Real-World Data
SUS+	Secondary Uses Service
SAE	Serious Adverse Events
SAR	Serious Adverse Reaction
SARS-COV-2	Severe Acute Respiratory Syndrome Coronavirus 2
SGLT2	Sodium-glucose co-transporter-2
SUSAR	Suspected Unexpected Serious Adverse Reactions
SWAT	Study Within A Trial
UK	United Kingdom

For peer review only - http://bmjopen.bmj.com/site/about/guidelines.xhtml

Trial acronym	Trial name
ASCEND	A Study of Cardiovascular Events iN Diabetes)
COPE	Characterisation of COPD Exacerbations using Environmental Exposure Modelling
DECIDE	Pragmatic Randomised 104 Week Multicentre Trial to Evaluate the Comparative Effectiveness of dapagliflozin and Standard of Care in Type-2 Diabetes
INTERVAL	(Interval is not an acronym)
RECOVERY	Randomised Evaluation of COVID-19 Therapy
REDUCE	(Not an acronym)
SANAD-II	A study of Standard and New Antiepileptic Drugs
StatinWise	Statin web-based investigation of side effects

For peer review only

For peer review only - http://bmjopen.bmj.com/site/about/guidelines.xhtml

REFERENCES

1. Recovery Collaborative Group, Horby P, Lim WS, et al. Dexamethasone in Hospitalized Patients with Covid-19 - Preliminary Report. *N Engl J Med* 2020.

2. Office for Life Sciences. Life sciences: industrial strategy. 2017. https://www.gov.uk/government/publications/life-sciences-industrial-strategy (accessed 2020-11-04.

3. NHS Digital. Transforming health and care through technology. 2020. https://digital.nhs.uk/about-nhs-digital/our-work/transforming-health-and-care-through-technology (accessed 2020-08-11.

4. National Institute of Health Research (NIHR). NIHR call for data-enabled trials. 2019. https://www.nihr.ac.uk/funding-and-support/funding-opportunities/data-enabled-trials/9417.

5. Clinical Practice Research Datalink. Interventional Studies. 2020. https://www.cprd.com/interventional-studies (accessed 2020-09-12.

6. NHS Digital, HDRUK DigiTrials Hub. https://digital.nhs.uk/services/nhs-digitrials (accessed 2020-07-09.

7. Heatlh Data Research UK. Pioneering data research centres to enable cutting-edge research and innovation to benefit UK patients. 2019. https://www.hdruk.ac.uk/news/pioneering-data-research-centres-to-enable-cutting-edge-research-and-innovation/ (accessed 2020-08-11.

8. Quint JK, Moore E, Lewis A, et al. Recruitment of patients with Chronic Obstructive Pulmonary Disease (COPD) from the Clinical Practice Research Datalink (CPRD) for research. *NPJ Prim Care Respir Med* 2018; **28**(1): 21.

9. Walters SJ, Bonacho Dos Anjos Henriques-Cadby I, Bortolami O, et al. Recruitment and retention of participants in randomised controlled trials: a review of trials funded and published by the United Kingdom Health Technology Assessment Programme. *BMJ open* 2017; **7**(3): e015276.

10. National Institute of Health Research (NIHR). Primary Care Specialty Profile: Why you should deliver primary care research in the UK. 2020. https://www.nihr.ac.uk/documents/primary-care-specialty-profile/11603 (accessed 2020-09-12.

11. Gulliford MC, Prevost AT, Charlton J, et al. Effectiveness and safety of electronically delivered prescribing feedback and decision support on antibiotic use for respiratory illness in primary care: REDUCE cluster randomised trial. *BMJ* 2019; **364**: l236.

12. Gulliford MC, Juszczyk D, Prevost AT, et al. Electronically delivered interventions to reduce antibiotic prescribing for respiratory infections in primary care: cluster RCT using electronic health records and cohort study. *Health technology assessment* 2019; **23**(11): 1-70.

13. NHS Scotland. SHARE. https://www.registerforshare.org/ (accessed 2019-07-03.

14. NHS. Coronavirus vaccine research. 2020. https://www.nhs.uk/conditions/coronavirus-covid-19/research/coronavirus-vaccine-research/ (accessed 2020-10-23.

15. Di Angelantonio E, Thompson SG, Kaptoge S, et al. Efficiency and safety of varying the frequency of whole blood donation (INTERVAL): a randomised trial of 45 000 donors. *The Lancet* 2017; **390**(10110): 2360-71.

16. Lai YS, Afseth JD. A review of the impact of utilising electronic medical records for clinical research recruitment. *Clin Trials* 2019; **16**(2): 194-203.

For peer review only - http://bmjopen.bmj.com/site/about/guidelines.xhtml

17. Murphy ME, Byrne M, Boland F, et al. Supporting general practitioner-based care for poorly controlled type 2 diabetes mellitus (the DECIDE study): feasibility study and protocol for a pilot cluster randomised controlled trial. *Pilot Feasibility Stud* 2018; **4**: 159.

18. clinicaltrials.gov. A Randomized Trial Assessing the Effects of Inclisiran on Clinical Outcomes Among People With Cardiovascular Disease (ORION-4). 2018. https://clinicaltrials.gov/ct2/show/NCT03705234 (accessed 2020-08-11.

19. Ascend Study Collaborative Group, Bowman L, Mafham M, et al. Effects of Aspirin for Primary Prevention in Persons with Diabetes Mellitus. *N Engl J Med* 2018; **379**(16): 1529-39.

20. Ascend Study Collaborative Group. ASCEND: A Study of Cardiovascular Events iN Diabetes. https://clinicaltrials.gov/ct2/show/NCT00135226 (accessed 2019-07-03.

21. Vestbo J, Leather D, Diar Bakerly N, et al. Effectiveness of Fluticasone Furoate-Vilanterol for COPD in Clinical Practice. *N Engl J Med* 2016; **375**(13): 1253-60.

22. Woodcock A, Vestbo J, Bakerly ND, et al. Effectiveness of fluticasone furoate plus vilanterol on asthma control in clinical practice: an open-label, parallel group, randomised controlled trial. *The Lancet* 2017; **390**(10109): 2247-55.

23. World Health Organisation. Specifications for Pharmaceutical Preparations -- Annex 5: Guidance on good data and record management practices. 2014. https://www.who.int/medicines/publications/pharmprep/WHO_TRS_996_annex05.pdf (accessed 2020-10-23.

24. Public Health England. Simulacrum: artificial patient-like cancer data to help researchers gain insights. https://healthdatainsight.org.uk/project/the-simulacrum/ (accessed 2019-07-03.

25. Health Data Research Innovation Gateway. Health Data Research Innovation Gateway: Explore datasets, tools and resources used in health research across the UK. 2020. https://www.healthdatagateway.org/ (accessed 2020-08-11.

26. Ahmed S, Sydes M, Love S, Carpenter J. Assessing the agreement of routinely-collected health data and case record form data in randomised controlled trials: a systematic review (CRD42020186048). 2020. https://www.crd.york.ac.uk/prospero/display_record.php?ID=CRD42020186048.

27. Sydes MR, Love SB, Murray ML, Lensen S, Macnair A, Carpenter J. SWAT 125: Comparison of trial-collected and routinely-collected death data. Queens University Belfast, 2020

28. Powell GA, Bonnett LJ, Tudur-Smith C, Hughes DA, Williamson PR, Marson AG. Using routinely recorded data in the UK to assess outcomes in a randomised controlled trial: The Trials of Access. *Trials* 2017; **18**(1): 389.

29. University of Liverpool, National Institute of Health Research (NIHR). Trial comparing the effectiveness and cost effectiveness of levetiracetam and zonisamide versus standard treatments for epilepsy: a comparison of Standard And New Antiepileptic Drugs (ISRCTN30294119). http://www.sanad2.org.uk/ (accessed 2019-07-03.

30. Pate A, Barrowman M, Webb D, et al. Study investigating the generalisability of a COPD trial based in primary care (Salford Lung Study) and the presence of a Hawthorne effect. *BMJ Open Respir Res* 2018; **5**(1): e000339.

31. Lensen S, Macnair A, Love SB, et al. Access to routinely collected health data for clinical trials - review of successful data requests to UK registries. *Trials* 2020; **21**(1): 398.

For peer review only - http://bmjopen.bmj.com/site/about/guidelines.xhtml

32. McKay AJ, Jones AP, Gamble CL, Farmer AJ, Williamson PR. Use of routinely collected data in a UK cohort of publicly funded randomised clinical trials. *F1000Research* 2020; **9**(323): 323.

33. NHS England. Data quality improvement. https://www.england.nhs.uk/data-services/validate/ (accessed 2020-08-11.

34. UK Health Data Research Alliance. UK Health Data Research Alliance. https://www.hdruk.ac.uk/digital-innovation-hubs/uk-health-data-research-alliance/ (accessed 2019-07-14.

35. Paprica PA, Sydes M, McGrail K, Morris A, Schull MJ, Walker R. Prospective data linkage to facilitate COVID-19 trials – A call to action. *International Journal of Population Data Science* 2020; **5**(2).

36. Chalmers I, Glasziou P. Avoidable waste in the production and reporting of research evidence. *Lancet* 2009; **374**(9683): 86-9.

37. Djulbegovic B, Kumar A, Glasziou P, Miladinovic B, Chalmers I. Medical research: Trial unpredictability yields predictable therapy gains. *Nature* 2013; **500**(7463): 395-6.

38. Glasziou P, Chalmers I. Research waste is still a scandal-an essay by Paul Glasziou and Iain Chalmers. *BMJ* 2018; **363**: k4645.

39. Department of Health and Social Care, Hancock MP M. NHS recovery to put staff wellbeing at its heart. 2020. https://www.gov.uk/government/news/nhs-recovery-to-put-staff-wellbeing-at-its-heart (accessed 2020-08-11.

40. Fogel DB. Factors associated with clinical trials that fail and opportunities for improving the likelihood of success: A review. *Contemp Clin Trials Commun* 2018; **11**: 156-64.

41. Getz KA, Zuckerman R, Cropp AB, Hindle AL, Krauss R, Kaitin KI. Measuring the Incidence, Causes, and Repercussions of Protocol Amendments. *Drug Information Journal* 2011; **45**(3): 265-75.

42. Clark LT, Watkins L, Pina IL, et al. Increasing Diversity in Clinical Trials: Overcoming Critical Barriers. *Curr Probl Cardiol* 2019; **44**(5): 148-72.

43. Marum RJ. Underrepresentation of the elderly in clinical trials, time for action. *British Journal of Clinical Pharmacology* 2020; **86**(10): 2014-6.

44. Bower P, Grigoroglou C, Anselmi L, et al. Is health research undertaken where the burden of disease is greatest? Observational study of geographical inequalities in recruitment to research in England 2013-2018. *BMC Med* 2020; **18**(1): 133.

45. National Institute of Health Research. The Include Ethnicity Framework. 2020. https://www.trialforge.org/trial-forge-centre/include/ (accessed 2020-11-19.

46. Hummers-Pradier E, Bleidorn J, Schmiemann G, et al. General practice-based clinical trials in Germany - a problem analysis. *Trials* 2012; **13**: 205.

47. Hummers-Pradier E, Scheidt-Nave C, Martin H, Heinemann S, Kochen MM, Himmel W. Simply no time? Barriers to GPs' participation in primary health care research. *Fam Pract* 2008; **25**(2): 105-12.

48. Medical Research Council (MRC), National INstitute of Health Research (NIHR). MRC-NIHR Trials Methodology Research Partnership (TMRP). 2019. https://www.methodologyhubs.mrc.ac.uk/about/tmrp/ (accessed 2019-07-03.

For peer review only - http://bmjopen.bmj.com/site/about/guidelines.xhtml

49. Treweek S, Bevan S, Bower P, et al. Trial Forge Guidance 1: what is a Study Within A Trial (SWAT)? *Trials* 2018; **19**(1): 139.

50. Treweek S. Why the NIHR's new funding stream for 'Studies Within A Trial (SWATs) is potentially game-changing. 2018.

51. MRC Hubs for Trials Methodology Research. SWAT Repository Store https://www.qub.ac.uk/sites/TheNorthernIrelandNetworkforTrialsMethodologyResearch/SWATSWARInformation/Repositories/SWATStore/ (accessed 2020-09-13.

52. Barry SJ, Dinnett E, Kean S, Gaw A, Ford I. Are routinely collected NHS administrative records suitable for endpoint identification in clinical trials? Evidence from the West of Scotland Coronary Prevention Study. *PLoS One* 2013; **8**(9): e75379.

53. The West of Scotland Coronary Prevention Study Group. Computerised record linkage: compared with traditional patient follow-up methods in clinical trials and illustrated in a prospective epidemiological study. *J Clin Epidemiol* 1995; **48**(12): 1441-52.

54. South A, Bailey J, Parmar MKB, Vale CL. Effectiveness and acceptability of methods of communicating the results of clinical research to lay and professional audiences: protocol for a systematic review. *Syst Rev* 2019; **8**(1): 150.

55. South A, Hanley B, Gafos M, et al. Models and impact of patient and public involvement in studies carried out by the Medical Research Council Clinical Trials Unit at University College London: findings from ten case studies. *Trials* 2016; **17**: 376.

56. South A, UCL MCTUa. Show results to participants engaged in clinical trials. 2019. http://www.isrctn.com/ISRCTN96189403.

57. Herrett E, Williamson E, Beaumont D, et al. Study protocol for statin web-based investigation of side effects (StatinWISE): a series of randomised controlled N-of-1 trials comparing atorvastatin and placebo in UK primary care. *BMJ open* 2017; **7**(12): e016604.

58. Parmar MK, Barthel FM, Sydes M, et al. Speeding up the evaluation of new agents in cancer. *J Natl Cancer Inst* 2008; **100**(17): 1204-14.

59. National Institute of Health Research (NIHR). NIHR Journals Library: Information for Authors. 2019.

60. Rothnie KJ, Mullerova H, Hurst JR, et al. Validation of the Recording of Acute Exacerbations of COPD in UK Primary Care Electronic Healthcare Records. *PLoS One* 2016; **11**(3): e0151357.

For peer review only - http://bmjopen.bmj.com/site/about/guidelines.xhtml

CONTRIBUTIONS

Organised workshop: M Landray, A Morris, J Valentine, R Walker, H Williams

Attended workshop: Y Barbachano, L Bowman, T Denwood, A Farmer, S Garfield-Birkbeck, M Gibson, M Gulliford, D Harrison, C Hewitt, M Landray, J Logue, A Morris, W Navaie, J Norrie, M O'Kane, J Quint, J Rycroft-Malone, J Sheffield, L Smeeth, F Sullivan, M Sydes, J Tizzard, J Valentine, R Walker, J Wilding, H Williams, P Williamson

Contributed to workshop discussion: Y Barbachano, L Bowman, T Denwood, A Farmer, S Garfield-Birkbeck, M Gibson, M Gulliford, D Harrison, C Hewitt, M Landray, J Logue, A Morris, W Navaie, J Norrie, M O'Kane, J Quint, J Rycroft-Malone, J Sheffield, L Smeeth, F Sullivan, M Sydes, J Valentine, R Walker, J Wilding, H Williams, P Williamson

Gave plenary presentation: L Bowman, T Denwood, M Gibson, M Gulliford, M Landray, M O'Kane, J Quint, L Smeeth, J Valentine, J Wilding, P Williamson

Chaired session: A Farmer, M Landray, A Morris

Chaired discussion group: D Harrison, C Hewitt, J Logue, J Norrie, F Sullivan, P Williamson

Member of discussion panel: Y Barbachano, W Navaie, J Rycroft-Malone, J Sheffield, J Tizzard

Scribed workshop: S Garfield-Birkbeck, M Sydes

Scribed discussion group: D Harrison, C Hewitt, J Logue, J Norrie, F Sullivan

Led writing of manuscript: M Sydes, J Valentine

Wrote key sections of manuscript: M Landray, M O'Kane, M Sydes, J Valentine, P Walker, R Walker, P Williamson

For peer review only - http://bmjopen.bmj.com/site/about/guidelines.xhtml

Contributed to and agreed manuscript: Y Barbachano, L Bowman, T Denwood, A Farmer, S Garfield-Birkbeck, M Gibson, M Gulliford, D Harrison, C Hewitt, M Landray, J Logue, A Morris, W Navaie, J Norrie, M O'Kane, J Quint, J Rycroft-Malone, J Sheffield, L Smeeth, F Sullivan, M Sydes, J Valentine, P Walker, R Walker, J Wilding, H Williams, P Williamson,

The Data Enabled Trials Group Workshop Group members (name, ORCID [if known], affiliation) are:

Deborah Ashby (0000-0003-3146-7466) [Imperial College London]; **Yolanda Barbachano** [Medicines and Healthcare products Regulatory Agency (MHRA)]; **Susan Beatty** [Clinical Practice Research Datalink]; **Marion Bennie** [HDR Scotland]; **Helen Bodmer** [Medical Research Council]; **Louise Bowman** (0000-0003-1125-8616) [Clinical Trial Service Unit and Epidemiological Studies Unit, Nuffield Department of Population Health, University of Oxford, Oxford]; **Emer Brady** (0000-0002-4715-9145) [Leicester General Hospital]; **Paul Brocklehurst** [North Wales Organisation for Randomised Trials]; **Brooke Jackson** [Clinical Practice Research Datalink]; **Marion Campbell** (0000-0001-5386-4097) [HDR Scotland]; **Andrea Cipriani** (0000-0001-5179-8321) [University of Oxford]; **Jane Daniels** (0000-0003-3324-6771) [University of Nottingham]; **Tom Denwood (**0000-0002-7337-2425) [NHS Digital]; **Michael Donnelly** [Queen's University Belfast]; **Tobias Dreischulte** [University of Dundee]; **Mark Edwards** (0000-0002-8616-3753) [University Hospital Southampton NHSFT]; **Andrew Farmer** (0000-0002-6170-4402) [Nuffield Department of Primary Health Care Sciences, University of Oxford.]; **Nick Freemantle** (0000-0001-5807-5740) [Comprehensive Clinical Trials Unit, UCL]; **Steph Garfield-Birkbeck** (0000-0002-3181-2840) [National Institute for Health Research Evaluation, Trials and Studies Coordinating Centre]; **Martin Gibson** (0000-0002-1331-1524) [North West E-Health (NWEH) Ltd]; **Steve Goodacre** (0000-0003-0803-8444) [National Institute of Health Research Health Technology Assessment CET**]; Doug Gould** (0000-0003-4148-3312) [Intensive Care National Audit & Research Centre (ICNARC)]; **Laura Gray** (0000-0002-9284-9321) [University of Leicester]; **Xavier Griffin** (0000-0003-2976-7523) [HDR UK Oxford]; **Martin Gulliford** (0000-0003-1898-9075) [Kings College London, London]; **David Harrison (**0000-0002-9002-9098) [Intensive Care National Audit & Research Centre (ICNARC), London, UK]; **Catherine Hewitt** (0000-0002-0415-3536) [York Trials Unit, Department of Health Sciences, University of York]; **Thomas Hiemstra** (0000-0002-2115-8689) [University of Cambridge]; **Anna Higgins** [University of Nottingham]; **Julia Hippisley-Cox** (0000-0002-2479-7283) [University of Oxford]; **Michael King** (0000-0003-4715-7171) [PRIMENT Clinical Trials Unit, UCL]; **Sasha Korniak** [Intensive Care

For peer review only - http://bmjopen.bmj.com/site/about/guidelines.xhtml

National Audit & Research Centre (ICNARC)]; **Martin Landray** (0000-0001-6646-827X) [Nuffield Department of Population Health, University of Oxford, Oxford, UK; Health Data Research UK, University of Oxford, Oxford, UK; National Institute of Health Research Oxford Biomedical Research Centre, Oxford University Hospitals NHS Foundation Trust, Oxford, UK]; **Martyn Lewis** (0000-0002-3667-132X) [Keele Clinical Trials Unit]; **Fiona Lobban** (0000-0001-6594-4350) [Lancaster University]; **Jennifer Logue** (0000-0001-9549-2738) [Lancaster University]; **Guillermo Lopez Campos** (0000-0003-3011-0940) [Queen's University Belfast]; **Stephanie MacNeill** [University of Bristol]; **Marion Mafham** (0000-0003-0562-3963) [HDR UK Oxford]; **Colin McCowan** (0000-0002-9466-833X) [HDR UK Scotland]; **Kathleen Meeley** [(Previously) Medicines and Healthcare products Regulatory Agency (MHRA)]; **Agnieszka Michael** (0000-0002-7262-6227) [Surrey Trials Unit]; **Nick Mills** [BHF Centre for Cardiovascular Sciences]; **Andrew Morris** (0000-0002-1766-0473) [Health Data Research UK]; **Will Navaie** [Health Research Authority]; **Irwin Nazareth** (0000-0003-2146-9628) [PRIMENT Clinical Trials Unit, UCL]; **Chris Newby** [Queen Mary University of London]; **John Norrie** (0000-0001-9823-9252) [Edinburgh Clinical Trials Unit, University of Edinburgh]; **Jacqui Nuttall** [Southampton Clinical Trials Unit]; **Martin O'Kane** [Medicines and Healthcare products Regulatory Agency (MHRA)]; **Jennifer Quint** (0000-0003-0149-4869) [NHLI, Imperial College London, UK]; **Dheeraj Rai** (0000-0002-7239-3523) [University of Bristol]; **Ian Roberts** (0000-0003-1596-6054) [London School of Hygiene & Tropical Medicine Clinical Trials Unit]; **Mike Robling** (0000-0002-1004-036X) [University of Cardiff**]; Jo Rycroft-Malone** (0000 0003 3858 5625) [Lancaster University & National Institute of Health Research Health Services & Delivery Programme]; **Jonathan Sheffield** [Formerly CEO National Institute of Health Research Clinical Research Network; University of Leeds]; **Aziz Sheikh** (0000-0001-7022-3056) [HDR UK (Scotland)]; **Murali Shyamsundar** (0000-0003-3797-8080) [Queen's University Belfast]; **Liam Smeeth** (0000-0002-9168-6022) [London School of Hygiene and Tropical Medicine]; **Claire Snowdon** [ICR Clinical Trials & Statistics Unit (ICR-CTSU)]; **Jamie Soames** (0000-0001-8948-3480) [National Institute of Health Research]; **Derek Stewart** [PPI]; **Adam Streeter** [University of Plymouth]; **Frank Sullivan** (0000-0002-6623-4964) [Division of Population & Behavioural Science, University of St. Andrews; Department of Family & Community Medicine, University of Toronto]; **Matthew Sydes** (0000-0002-9323-1371) [MRC Clinical Trials Unit at UCL, Institute of Clinical Trials and Methodology, UCL, London, UK & HDR UK]; **Doreen Tembo** (0000-0002-8615-3563) [National Institute of Health Research Evaluation, Trials and Studies Coordinating Centre (NETSCC)]; **Juliet Tizzard** [Health Research Authority]; **Catrin Tudur Smith** (0000-0003-3051-1445) [University of Liverpool]; **Janet Valentine** [Clinical Practice Research Datalink]; **Paula Walker** [Medicines and Healthcare products Regulatory Agency (MHRA)]; **Rhoswyn**

For peer review only - http://bmjopen.bmj.com/site/about/guidelines.xhtml

Walker (0000-0001-5605-2879) [Health Data Research UK]; **Alan Watkins** [University of Swansea]; **Will Whiteley** (0000-0003-2839-8404) [Department of Cardiovascular and Metabolic Medicine; Institute of Life Course and Medical Sciences; University of Liverpool]; **John Wilding** (0000-0003-2839-8404) [Department of Cardiovascular and Metabolic Medicine, Institute of Life Course and Medical Sciences, University of Liverpool]; **Hwyel Williams** (0000-0002-5646-3093) [Professor of Dermato-Epidemiology, University of Nottingham]; **Kate Williams** [University of Cambridge]; **Elizabeth Williamson** (0000-0001-6905-876X) [London School of Hygiene & Tropical Medicine]; **Paula Williamson** (0000-0001-9802-6636) [University of Liverpool]

For peer review only

For peer review only - http://bmjopen.bmj.com/site/about/guidelines.xhtml

COMPETING INTERESTS

J Wilding reports grants, personal fees and other from AstraZeneca, other from Astellas, other from Boehringer Ingelheim, other from Lilly, other from Napp, grants, personal fees and other from Novo Nordisk, other from Sanofi, grants, personal fees and other from Takeda, other from Rhythm Pharma, outside the submitted work.

The other authors do not declare relevant competing interests for this work.

For peer review only
